# Male courtship song drives escape responses that are suppressed for successful mating

**DOI:** 10.1038/s41598-021-88691-w

**Published:** 2021-04-29

**Authors:** Eliane Arez, Cecilia Mezzera, Ricardo M. Neto-Silva, Márcia M. Aranha, Sophie Dias, Marta A. Moita, Maria Luísa Vasconcelos

**Affiliations:** 1 Champalimaud Research, Champalimaud Center for the Unknown, 1400-038 Lisbon, Portugal; 2 Trinity College Institute of Neuroscience, School of Genetics and Microbiology, Smurfit Institute of Genetics and School of Natural Sciences, Trinity College Dublin, Dublin-2, Ireland

**Keywords:** Genetics of the nervous system, Neural circuits, Sexual behaviour

## Abstract

Persuasion is a crucial component of the courtship ritual needed to overcome contact aversion. In fruit flies, it is well established that the male courtship song prompts receptivity in female flies, in part by causing sexually mature females to slow down and pause, allowing copulation. Whether the above receptivity behaviours require the suppression of contact avoidance or escape remains unknown. Here we show, through genetic manipulation of neurons we identified as required for female receptivity, that male song induces avoidance/escape responses that are suppressed in wild type flies. First, we show that silencing 70A09 neurons leads to an increase in escape, as females increase their walking speed during courtship together with an increase in jumping and a reduction in pausing. The increase in escape response is specific to courtship, as escape to a looming threat is not intensified. Activation of 70A09 neurons leads to pausing, confirming the role of these neurons in escape modulation. Finally, we show that the escape displays by the female result from the presence of a courting male and more specifically from the song produced by a courting male. Our results suggest that courtship song has a dual role, promoting both escape and pause in females and that escape is suppressed by the activity of 70A09 neurons, allowing mating to occur.

## Introduction

Mating rituals serve many different purposes, such as attracting potential mates, synchronizing reproduction, announcing the animal’s species, sex and fitness, persuading the mate to overcome contact aversion^1^. A prospective mate that is unreceptive to the courtship advances will likely flee the scene^2^.

In *Drosophila melanogaster* courtship, the male performs a series of distinct and stereotyped motor programs such as orienting towards the female, following her while extending and vibrating one wing producing a courtship song, quivering the abdomen, tapping and licking female’s genitals and, finally, attempting copulation^3–5^. During male courtship the female exhibits behaviours that may be interpreted as rejection responses such as wing flicking, ovipositor extrusion, fending, decamping and kicking^6–9^. Although performed at different levels, rejection behaviours are displayed by both receptive and unreceptive females^6,10–13^ and constitute the means by which the female communicates with the male. Thus, receptive females are thought to temporarily reject the courting male to collect quantitative and qualitative information about him^3,9,11,14^. Despite the mild rejections, a receptive female will eventually slow down and open the vaginal plates to induce the male to copulate^6,8,12,15^. Female locomotor activity is tightly coupled with receptivity since unreceptive flies (either sexually immature, mated, or manipulated) do not slow down nor pause as much as receptive females^6,8,16–21^. More specifically, receptive females slow down in response to the male’s courtship song^17–19,21–24^. The relationship between locomotor activity and song has been mechanistically explored in recent years. Besides auditory neurons^22,24–27^, the higher order pC2 neurons are involved in the regulation of locomotion upon song presentation^23^, as indicated by the negative correlation of speed and calcium responses of female pC2 neurons to a song stimulus. Genetic manipulation of pC2 activity indicates that other circuit elements must contribute to the locomotor tuning for the song, since activation of pC2 neurons leads to multiphasic speed responses and their silencing leads to a correlation between speed and the interpulse interval of the song which is uncorrelated in wild type females. pC1 neurons, which integrate multiple inputs such as internal sensing of the mating status^28^ and the male pheromone cis-vaccenyl acetate^29^, also respond to song^29^, though how these contribute to a locomotor response has not been shown.

With the goal of understanding the behavioural and neuronal mechanisms of female receptivity, we combined detailed quantitative description of female behaviour during courtship with neuronal manipulations. These approaches inform each other. While detailed behavioural analysis constitutes a window into brain function as it allows the mapping of specific sets of neurons or circuits to specific behavioural outputs, the identification and manipulation of neurons involved in receptivity contribute to the dissection of the modular structure of receptivity. In a female receptivity screen, aimed at identifying brain neurons where higher order receptivity would take place, we identified a group of neurons (line 70A09) that, when silenced, render the female unreceptive. Specifically, when silencing the 70A09 neurons, sexually mature flies in the presence of a courting male walk faster, pause less and jump more than control flies, behaviours that are hallmarks of an escape response, which could explain why they are unreceptive. However, even if escape is impeded, they still did not mate. Furthermore, the increased escape response was specific to the courtship context, as these flies did not increase escape response triggered by general threats, such as a large overhead looming stimulus. Conversely, acutely activating 70A09 neurons lead to a halt in walking. We further confirmed the requirement of courtship to elicit the escape response by pairing 70A09-silenced females with males that do not court. Finally, we showed that the courtship song is key to elicit escape. In summary, we identified a new role of the male courtship song in eliciting female escape and a set of neurons in the female brain that are involved in suppressing such courtship song-induced escape response. We propose that the male song has a dual role, first eliciting escape and providing the female with enough time to assess the male, until the decision to mate is made, upon which then the song prompts a decrease in locomotion and that activity in 70A09 neurons is necessary to suppress the initial song-induced escape.

## Results

### Silencing *70A09*-GAL4 brain neurons reduces female receptivity

**Figure 1:**
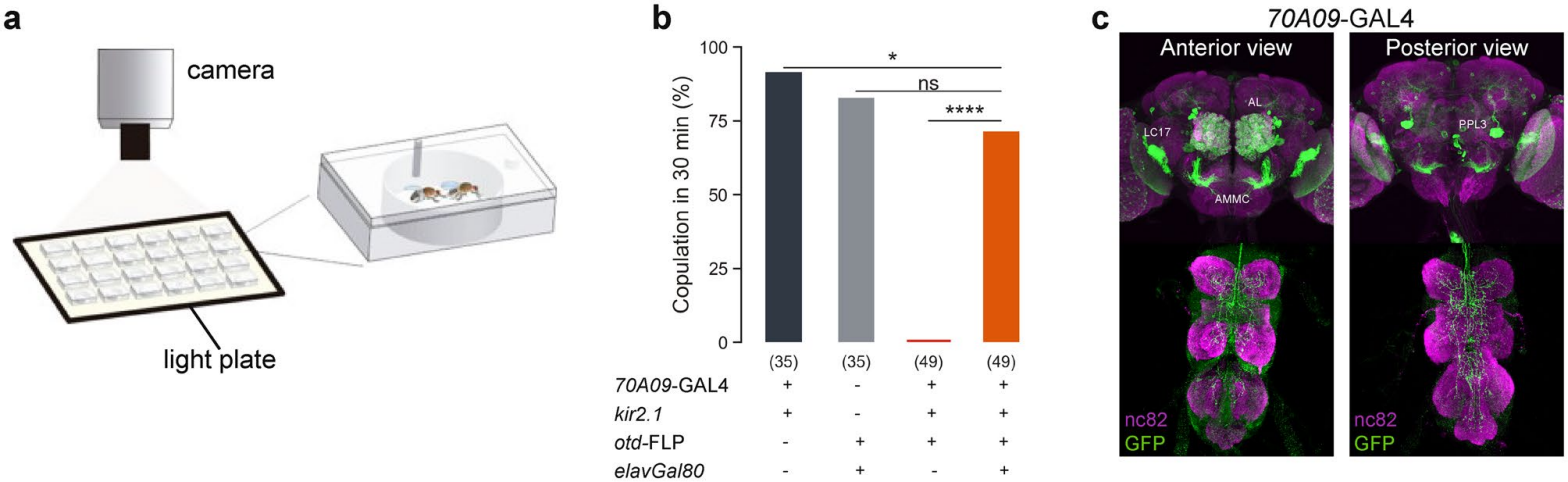
Silencing 70A09 brain neurons reduces female receptivity. (**a**) Schematic representation of the behavioural setup to test female receptivity^16^. Mating arena containing mating pairs is highlighted. (**b**) Copulation rate of silenced and control females with n values shown in parentheses. Statistical analysis was performed with Fisher’s exact test: ns = not significant; *p < 0.05, ****p < 0.0001. (**c**) Anterior and posterior views of female brain and VNC showing the expression pattern of 70A09-GAL4/otd-nls:FLPo intersecting neurons. Neurons were visualised with anti-GFP (green) and the tissue counterstained with the synaptic marker nc82 (magenta).

In order to identify neurons involved in female receptivity, we performed a silencing screen of the Janelia GAL4 line collection^30^. Silencing was achieved with the expression of an inward rectifier potassium channel, *Kir2.1*^31^, that reduces the probability for an action potential to occur by hyperpolarizing the neurons. To prevent developmental lethality, silencing was restricted to the adult stage using temperature sensitive GAL80^32^ which inhibits the expression of *Kir2.1*. The control flies have the same genotype but a different temperature treatment, though all flies were tested at 25ºC (see methods). We tested 1042 lines for fertility and identified 65 lines in which at least 25% of the silenced females did not produce progeny (n = 20–25). Next, we tested these lines for receptivity. For this, we paired a single wild type naïve male and a silenced virgin female in an arena and quantified copulation within 30 min (Fig. 1a). With this secondary screen we identified 20 lines that affected receptivity when silenced (Table S1). Finally, we selected eight lines based on the strength of the phenotype, absence of neurons known to affect receptivity, such as, sex-peptide sensing neurons^33–35^, and confirmation that the phenotype results from neuronal disruption using *elav-GAL80* (see below). We next retested these lines while restricting the neuronal manipulation to the brain using a flippase under the control of the orthodenticle promoter *(otd*)^36^. The lines 70A09 and 57G02 showed a marked reduction in copulation when brain neurons were silenced in the adult female (Figure S1). All statistical details related to main Figures and Supplementary Figure are shown in Tables S2 and S3, respectively. The line 70A09 was selected for further analysis considering the more restricted expression pattern when compared to 57G02 (data not shown). The loss of receptivity when silencing neurons labelled by the line 70A09 (Figure S1) was confirmed with constitutive silencing where no temperature treatment is applied (Fig. 1b). In this case, the controls are the two parental lines (lines used in the cross to obtain the test flies) crossed with the line w1118 which was the basis for the generation of all the transgenic lines in this work, therefore providing a neutral genetic background. Constitutive silencing was used in the subsequent experiments of this work because it is not lethal and it involves simpler and faster husbandry compared to conditional silencing. To confirm that the observed phenotype was a consequence of neuronal disruption we used *elav-GAL80*^34^ to prevent *Kir2.1* expression in neurons. We did not observe abolishment of receptivity in these females (Fig. 1b), indicating that the reduced receptivity is a result of neuronal silencing.

Immunostaining of the *70A09-GAL4* brain neurons revealed many neuronal groups that could play a role in female receptivity (Fig. 1c). To identify the neurons responsible for the receptivity phenotype observed, we used two approaches that involved intersections with 70A09. In both approaches, in-house generated splitGAL4 and a LexA version of 70A09 were used to allow for more flexibility in the intersections (Figure S2). One approach was to generate intersections that separately label each of the groups of neurons that can be identified in the immunostaining. Using this approach, we labelled and tested (i) the auditory sensory neurons (Figure S3a), (ii) the local GABAergic antennal lobe neurons (Figure S3b), (iii) neurons that express the insulin-like peptides in the pars intercerebralis (Figure S3c), (iv) the lobula columnar neurons (LC17) (Figure S3d) and (v) the protocerebral posterior lateral cluster (PPL3) (Figure S3e). None of the separate groups recapitulated the *70A09-GAL4* (from here on referred to as 70A09) silencing phenotype. The second approach was to intersect the 70A09 line with lines of genes involved in generating sexually differentiated circuits, *fruitless* (*fru*) and *doublesex* (*dsx*)^9^. Immunostaining of the intersection of 70A09 with *fru* shows labelling of local antennal neurons and auditory sensory neurons, corresponding to the GABAergic neurons of the 70A09 line (Figure S3f and S3b). Some additional labelling is observed in the protocerebrum corresponding to neurons located in the ventral nerve cord (VNC) that project to the brain since the intersection in this case is not restricted to the brain. The *fru* intersection line was not tested further since *fru*-positive brain neurons were shown in the first approach to not be involved in the receptivity phenotype (Figure S3a, b and f) and the *fru*-positive ascending neurons are out of the scope of this work. Silencing *dsx*-positive 70A09 (70A09⋂*dsx*) neurons does lead to a reduction of receptivity (Figure S3g). Immunostaining of this intersection (Figure S3g) showed labelling of pC1 neurons which had been shown to modulate receptivity^15,29^. In fact, the degree of reduction in receptivity resembled that observed by Zhou et al.^29^. However, this reduction in receptivity is partial and does not explain the complete abolishment of receptivity observed in the 70A09-silenced females hence other neurons must be involved. The candidates are smaller cells with diffuse innervation which remain untested.

In summary, 70A09 labels brain neurons involved in female receptivity which include but are not restricted to pC1 neurons.

### 70A09-silenced females escape in response to male courtship

**Figure 2:**
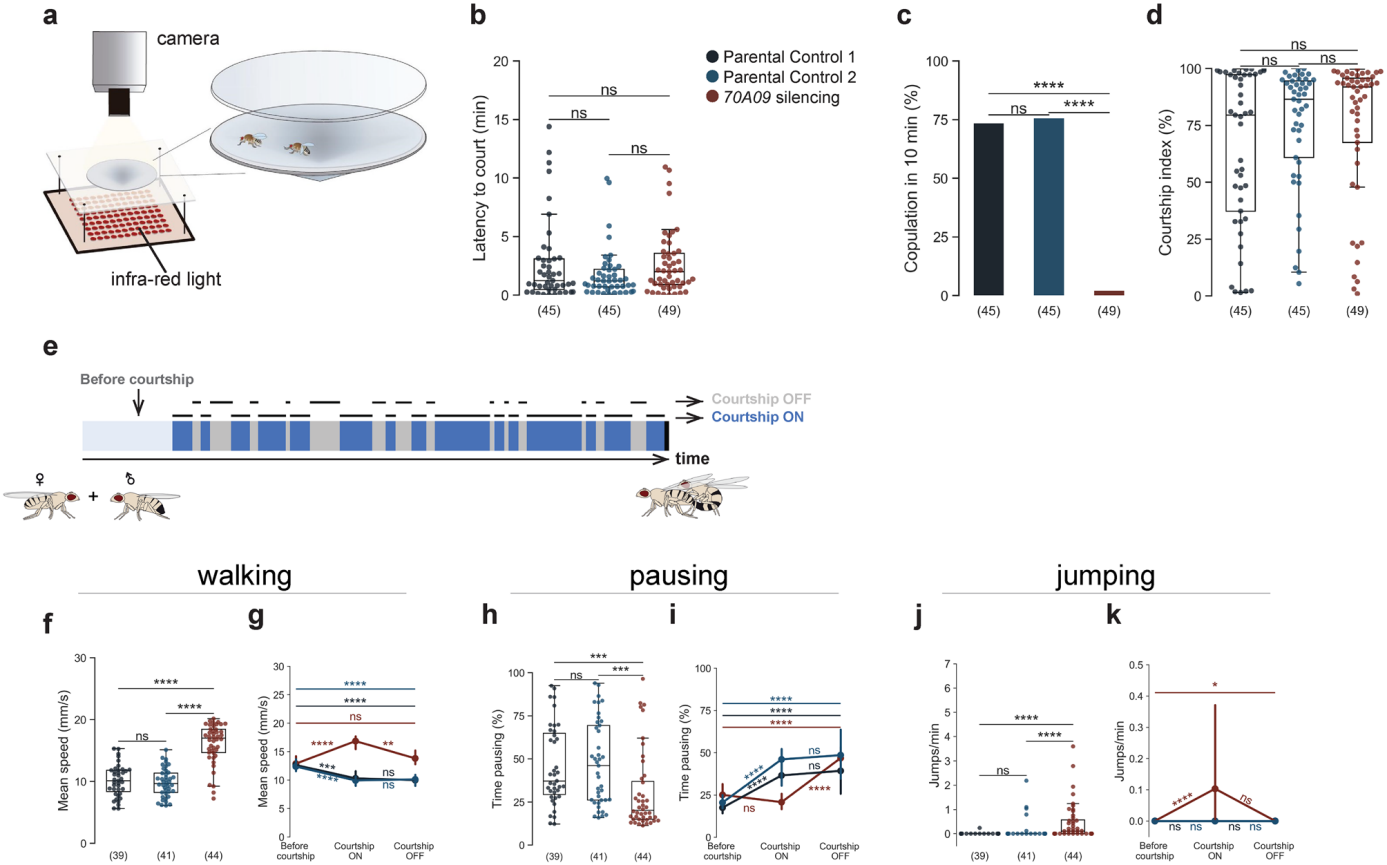
70A09-silenced females escape in response to male courtship. (**a**) Schematic representation of the behavioural setup to quantify and characterise receptivity behaviour^16^. (**b**) Male latency to court. Genotypes: w-/UAS > STOP > kir2.1; otd-nls:FLPo/ + ; + (Parental Control 1), w-; + ; 70A09-GAL4/ + (Parental Control 2) and w-/UAS > STOP > kir2.1; otd-nls:FLPo/ + ; 70A09-GAL4/ + (70A09 silencing). (**c**) Copulation rate of silenced and control females. (**d**) Courtship index toward silenced and control females. (**e**) Schematic representation of the male courtship dynamic: before courtship (period from the start of recording to the start of courtship), courtship ON (bouts of courtship) and courtship OFF (bouts of non-courtship). (**f**–**k**) Behavioural effects of silencing 70A09 brain neurons on female mean walking speed (4–50 mm/s) (**f** and **g**), female pausing (**h** and **i**) and number of jumps per minute (**j** and **k**), during courtship ON periods (**f**,**h** and **j**) or in different moments of courtship dynamics (**g**,**i** and **k**). Statistical analysis was performed with Fisher’s exact test (**c**), Kruskal–Wallis test (**b**,**d**,**f**,**h** and **j**) and Friedman’s test (**g:** parental control 1 and 70A09 silencing, **i** and **k**) followed by post hoc pairwise Dunn’s test with Bonferroni correction, repeated measures ANOVA followed by post hoc multiple pairwise paired t-test with Bonferroni correction (**g:** parental control 2): ns = not significant, *p < 0.05, **p < 0.01, ***p < 0.001, ****p < 0.0001. n values are shown in parentheses.

To characterise the behaviour of 70A09-silenced females during courtship, we now used a setup that allows tracking the flies (Fig. 2a). We analysed flies’ behaviours from the start of courtship up to 10 min or until copulation (in those cases where copulation occurred in less than 10 min). We recorded single pairs for 20 min or until copulation to account for variability in latency to court (Fig. 2b). First, we tested the female receptivity phenotype to validate the use of the setup. We observed that receptivity is also abolished in the arena with a different size, shape and lighting (Fig. 2c). To confirm that the reduced copulation rate is due to reduced receptivity rather than reduced attractiveness of the female, we measured the courtship elicited by these females. We observed that males take about the same time to initiate courtship and court at the same levels silenced and control females (Fig. 2b and d).

Female locomotor activity is one of the most reliable indicators of the female’s willingness to copulate^6,8,16–21^, therefore we measured walking speed and pausing levels. Given that courtship happens in bouts, we quantified walking speed in three distinct moments of courtship dynamics, represented in Fig. 2e: before courtship starts, during courtship (‘courtship ON’), and during intervals between courtship bouts (‘courtship OFF’). Quantification of walking speed during courtship ON revealed that 70A09-silenced females walk at a substantially higher speed than control females (Fig. 2f). It is known that unmanipulated females slow down during courtship^6,8,16–21^. Thus, the difference in walking speed during courtship could result from silenced females not responding to male courtship, i.e., not slowing down like control females. To address this, we compared walking speed during courtship ON with other moments. We observed that rather than sustaining the speed, 70A09-silenced females increase walking speed during courtship ON compared to before courtship (Fig. 2g). The increase in speed is acute since, during courtship OFF, 70A09-silenced females return to the walking speed exhibited before courtship. This observation is in sharp contrast with control females that reduce the walking speed during courtship ON and sustain this reduced speed during courtship OFF. Next, we analysed female pausing as it has been reported to increase during courtship^8,17^. We found that 70A09-silenced females pause less during courtship compared to control females (Fig. 2h). Comparing across different courtship moments we found that, contrary to courtship ON moments, pausing increases in courtship OFF (Fig. 2i). The increase in walking speed and reduced rest are means for the female to escape the male. A third way to escape the male is to take off in flight, which in an enclosed arena results in a jump. For this reason, we investigated whether jumping was affected in manipulated flies. Indeed, during courtship ON 70A09-silenced females jump more than control flies (Fig. 2j). Jumping in 70A09-silenced females is strongly increased during courtship ON compared to before courtship (Fig. 2k). During courtship OFF jumping decreases though not significantly, suggesting that the females remain aroused.

In the previous section we have shown that the receptivity phenotype was partially due to 70A09⋂*dsx* neurons. To test whether this subset of 70A09 neurons is also involved in the escape phenotype, we tested the 70A09⋂*dsx* silencing in the tracking setup. Analysis of walking speed, pausing and jumping shows that 70A09⋂*dsx*-silenced females do not escape a courting male (Figure S4). In other words, *dsx* neurons within the 70A09 line are not involved in the courtship-induced escape phenotype.

Altogether, our findings suggest that activity in 70A09 neurons is required for females to suppress escape responses during courtship.

### Silencing 70A09 neurons does not increase escape responses upon threat

**Figure 3:**
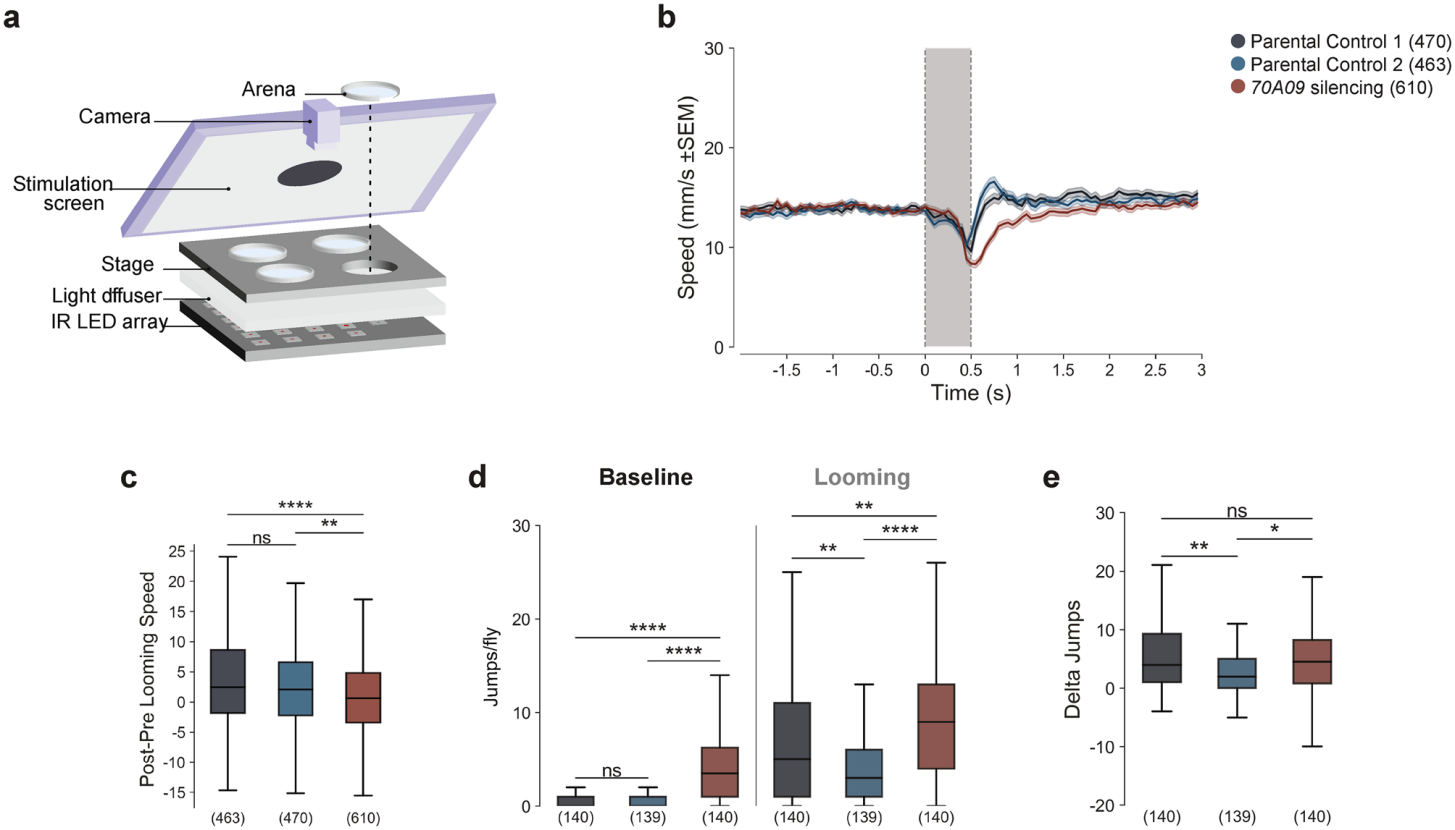
Silenced 70A09 females show less vigorous escape responses when exposed to a threat. (**a**) Schematic of the experimental setup used to characterise escape behaviours in response to looming stimuli^40^. Genotypes: + /w-, UAS > STOP > kir2.1; otd-nls:FLPo/ + ; + (Parental Control 1), + /w-; + ; 70A09 GAL4/ + (Parental Control 2) and + /w, UAS > STOP > kir2.1; otd-nls:FLPo/ + ; 70A09-GAL4/ + (70A09 silencing). In **b** and **c** only looming events where flies were walking before and after the stimulus were included. (**b**) Looming-triggered speed profile. Average (± SEM) speed in a time window around looming. Dashed lines indicate looming onset and offset. Shaded grey area indicates looming duration. (**c**) Change in speed caused by stimulus presentation (pre-looming period subtracted from post-looming period). (**d**) Number of jumps per fly during the baseline and stimulation period. (**e**) Increase in the number of jumps per fly during stimulation relative to baseline (jumps per fly during stimulation subtracted from jumps per fly during baseline). Center line, median; box limits, upper (75) and lower (25) quartiles; whiskers, 1.5 × interquartile range. Statistical analysis was performed with Kruskal–Wallis test, followed by post hoc pairwise Dunn’s test with Bonferroni correction: ns = not significant, *p < 0.05, **p < 0.01, ****p < 0.0001. n values are shown in parentheses and indicate the number of looming events in **b** and **c**, and the number of flies in **d** and **e**.

To determine if 70A09 neurons are involved in general escape responses, we tested the response of 70A09-silenced females to looming stimuli. When exposed to looming in an enclosed arena, fruit flies have been shown to display different defensive responses, namely freezing, running and jumping^37–40^. To analyse escape responses, i.e. running and jumping, of 70A09-silenced females, we adapted a previously established behavioural paradigm (Fig. 3a)^40^. Single flies were transferred to a covered arena and allowed 2 min to explore. This baseline period was followed by 5 min during which the flies were exposed to 7 repetitions of a looming stimulus, displayed on a computer monitor angled above the arenas (Fig. 3a).

To examine the profile of escape responses, we plotted the average speed of the flies aligned to looming onset (Fig. 3b). We found that the speed was constant and similar between unmanipulated and silenced flies before stimulus onset. Upon looming onset, flies showed a sharp decrease in their speed, which was followed by a rapid increase in locomotion that was less pronounced for silenced flies. The elevation in speed relative to that observed before looming onset was more noticeable for control flies than for 70A09-silenced females.

To better characterise this disparity in escape responses, we quantified the difference in speed (delta speed) between a defined time window (0.5 s) after looming offset and before looming onset (Fig. 3c). We found that the increase in speed in response to looming stimuli was significantly lower for 70A09-silenced females compared to controls. The less vigorous escape responses observed for silenced females in response to threat differ from what was observed in the context of courtship.

In response to a courting male, besides increased walking speed and reduced pausing, silenced flies also show an increase in jumps, that likely correspond to take-off attempts. Therefore, we also investigated jumping responses upon visual threat. We quantified the number of escape jumps per fly for the different genotypes during the baseline and stimulation periods (Fig. 3d). During the stimulation period, silenced females jumped significantly more than both controls. However, we found that during baseline silenced females also jumped significantly more than unmanipulated females. Given this result, we asked if the increase in jumps observed during stimulation relative to those observed in baseline was significantly higher for silenced females. For each genotype, we calculated the difference between the number of jumps observed during stimulation and baseline (delta jumps) (Fig. 3e), and we found a significant difference between the silenced condition and only one of the controls.

Together, these results indicate that the increased escape displayed by 70A09-silenced females in the context of courtship is a specific response to the courting male, not observable in a general threat context.

### 70A09-silenced females are unreceptive independently of escape availability

**Figure 4:**
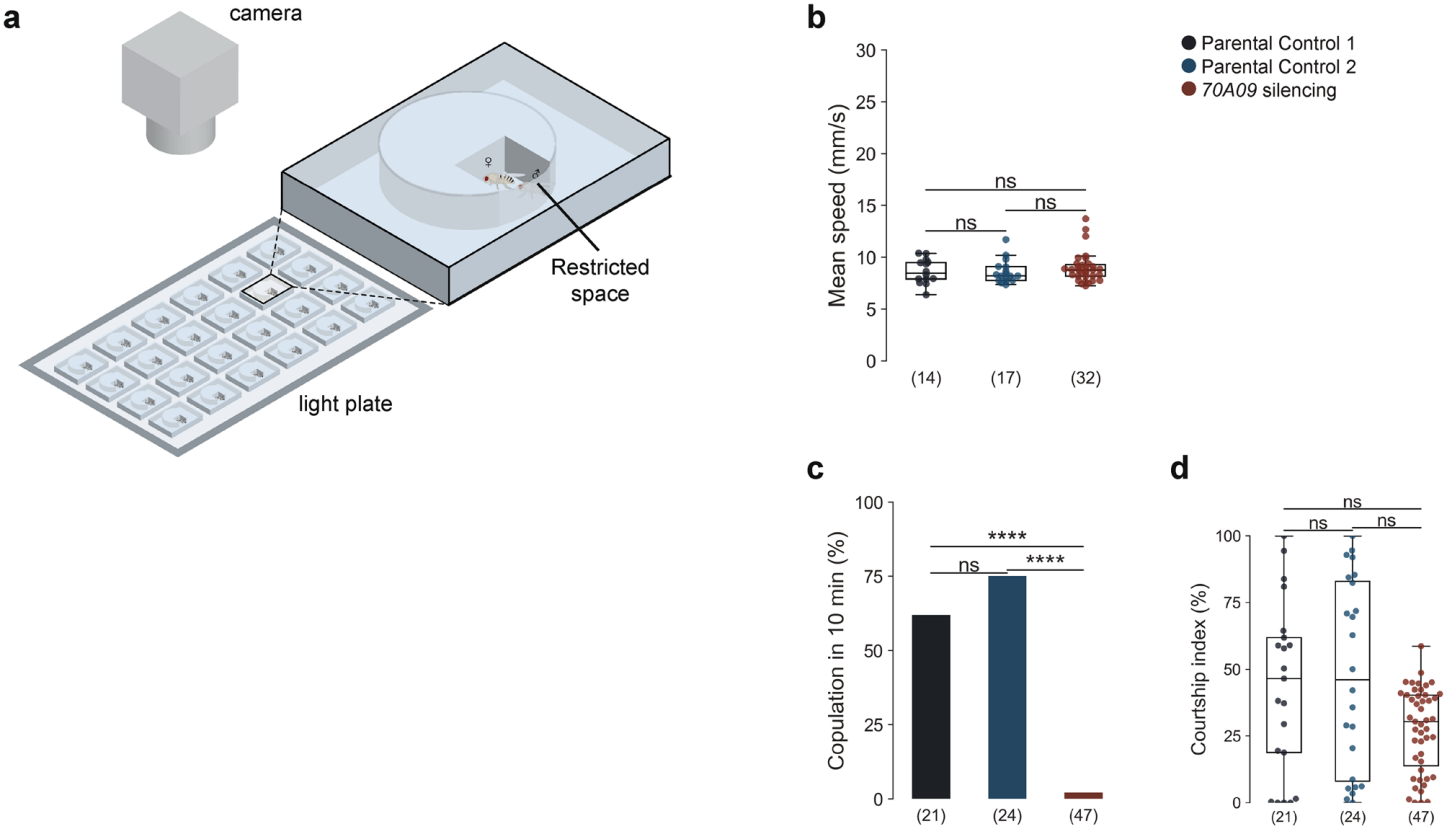
70A09-silenced females remain unreceptive when escape is impeded. (**a**) Schematic representation of the behavioural setup to test female receptivity when the female is not allowed to walk away from the male. Arena drawn by Gil Costa. (**b**) Female mean walking speed (4–50 mm/s) during courtship ON periods. Genotypes: w /UAS > STOP > kir2.1; otd-nls:FLPo/ + ; + (Parental Control 1), w-; + ; 70A09-GAL4/ + (Parental Control 2) and w-/UAS > STOP > kir2.1; otd-nls:FLPo/ + ; 70A09-GAL4/ + (70A09 silencing). (**c**) Copulation rate of silenced and control females. (**d**) Male courtship index toward silenced and control females. Statistical analysis was performed with Fisher’s exact test (**c**) and, Kruskal–Wallis test followed by post hoc pairwise Dunn’s test with Bonferroni correction (**b** and **d**): *ns* not significant, ****p < 0.0001. n values are shown in parentheses.

The increased walking speed of 70A09-silenced females during courtship raises the question of whether the absence of mating is merely a consequence of the inability to slow down. To address this question, we restricted the walking space of the arenas used for screening with the introduction of an adapter (restricted arenas, Fig. 4a). In this new version the space is 6 mm × 5 mm × 4.5 mm, which allows movement but not running (consider for reference that a fly is around 2 mm long). We paired single flies for 20 min and analysed for up to 10 min after courtship initiation. We confirmed that indeed in these arenas 70A09-silenced females do not speed up but rather walk at similar speed of control females (Fig. 4b). We found that, in this context, silenced females still did not mate (Fig. 4c). The male courtship index is similar in all conditions showing that the difference in copulation rate does not result from low male drive (Fig. 4d). In sum, our results show that 70A09 females are unreceptive independently of their ability to escape.

### Courtship, and specifically courtship song, is required for 70A09-dependent escape suppression

**Figure 5:**
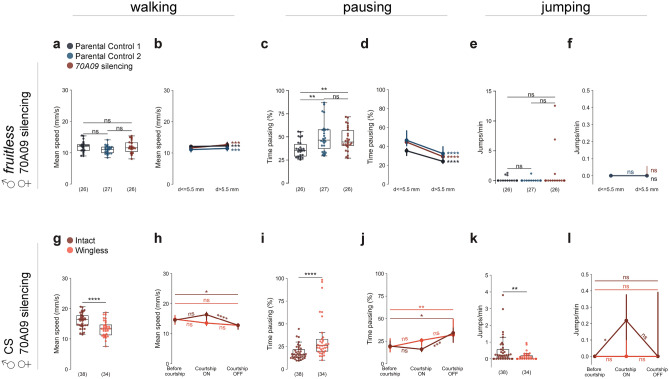
70A09-silenced females do not escape when coupled with courtship-impaired males. (**a**–**f**) Non courting fruitless mutant males paired with females of each of the genotypes: w-/UAS > STOP > kir2.1; otd nls:FLPo/ + ; + (Parental Control 1), w-; + ; 70A09-GAL4/ + (Parental Control 2) and w-/UAS > STOP > kir2.1; otd-nls:FLPo/ + ; 70A09-GAL4/ + (70A09 silencing). (**g**–**l**) Canton-S males intact and with wings removed (wingless) paired with silenced 70A09 females (70A09 silencing). (**a** and **g**) Female mean walking speed (4–50 mm/s), (**c** and **i**) female pausing and (**e** and **k**) number of jumps per minute, during courtship ON periods. (**b** and **h**) Female mean walking speed (4–50 mm/s), (**d** and **j**) female pausing and (**f ** and **l**) number of jumps per minute, in different moments of courtship dynamics. Statistical analysis was performed with one-way ANOVA followed by post hoc Tukey’s test (**a**), paired t-test (**b**), Kruskal–Wallis test (**c** and **e**) and Friedman’s test (**h**,**j** and **l**) followed by post hoc pairwise Dunn’s test with Bonferroni correction, Wilcoxon signed rank test (**d** and **f**), unpaired t-test (**g**) and Mann–Whitney U test (**i** and **k**): *ns* not significant, *p < 0.05, **p < 0.01, *** p < 0.001, ****p < 0.0001. n values are shown in parentheses.

To confirm that increase in walking speed, reduction in pausing and increase in jumps in 70A09-silenced females is a response to courtship we paired female flies with *fru* mutant males that do not court^41^. Since courtship was absent, we used the distance between the flies as a proxy for courtship as we have previously shown that below 5.5 mm there is a 95.5% likelihood of courtship (‘courtship distance’)^16^. We observed no difference between the walking speed of 70A09-silenced females and control females at courtship distance (Fig. 5a), as well as, no difference in walking speed between courtship distance and not courtship distance for all conditions (Fig. 5b). These results indicate that the changes in female walking speed require courtship from a male, the mere presence of a male not being sufficient to trigger them. Pausing levels were also very different from those observed in females paired with a courting male. At courtship distance, silenced flies pause either as much or more when compared to the parental controls (Fig. 5c). In all conditions there is more pausing at courtship distance (Fig. 5d). Finally, jumps were nearly absent in all conditions (Fig. 5e and f). From our results, we conclude that a courting male and not the mere presence of a male triggers escape in 70A09-silenced females.

A courting male produces different stimuli that may lead the female to escape. They could be the visual stimulus of an approaching animal, the scent of male pheromone or the song that the male produces with wing vibration. Given that song has been shown to modulate the speed of the female during courtship, albeit to reduce it^17,19,21,22,22–24^, we decided to test the role of song in the response of 70A09-silenced females. For this, we paired 70A09-silenced females with wild type males that were either intact or with the wings removed (‘wingless’). We first confirmed that courtship index is not affected by wing removal (Figure S5). We then analysed the female walking speed in the two different conditions. We found that, during courtship, the walking speed of 70A09-silenced females was lower for females paired with wingless males (Fig. 5g). During the different moments of courtship, the walking speed of females paired with wingless males never changed whereas control silenced females with intact males, as previously found (Fig. 2f), increased their walking speed during courtship ON moments compared to courtship OFF moments (Fig. 5h). In this experiment, however, the walking speed of 70A09-silenced females with intact males is not significantly different between baseline and courtship ON moments (Fig. 5h), unlike what was previously found (Fig. 2f), which may be a reflection of a trend for higher baseline walking speed observed in this experiment when compared to the previous experiment (Mann–Whitney U, U = 623, p = 0,0516). Analysis of pausing during courtship ON revealed that 70A09-silenced females paired with wingless males pause more than those paired with intact males (Fig. 5i). Across the different moments of courtship 70A09-silenced females paired with wingless males have similar pausing levels with a small increase of pausing in courtship OFF compared to before courtship (Fig. 5j). Finally, 70A09-silenced females paired with wingless males jump very little during courtship ON (Fig. 5k) or any other moment of the video (Fig. 5l) whereas 70A09-silenced females paired with intact males significantly increase jumps during courtship ON compared to before courtship with no significant difference in courtship OFF moments. These results clearly show that a courting male that is unable to produce song does not elicit any type of escape in 70A09-silenced females, i.e., that song is a trigger for escape in 70A09-silenced females.

### Activation of 70A09 neurons leads to female pausing but not mating

**Figure 6:**
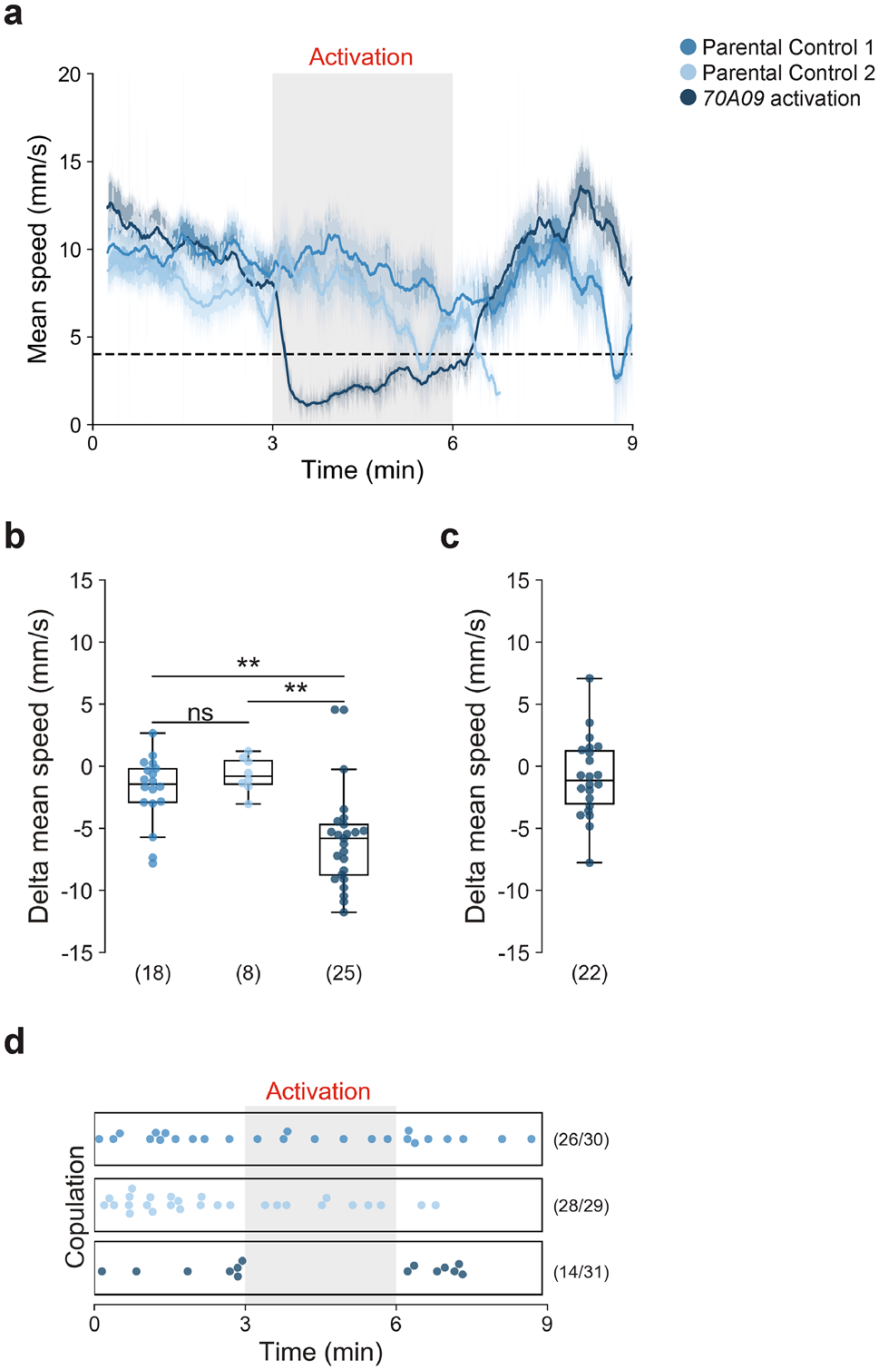
Activation of 70A09-GAL4 brain neurons drastically reduces female speed. (**a**) Female mean speed calculated by rolling average for 15 s with Standard Error of the Mean represented. Genotypes: w-; otd-nls:FLPo/ + ; UAS > STOP > Chrimson.mVenus (Parental Control 1), w-; + ; 70A09-GAL4/ + (Parental Control 2) and w-; otd-nls:FLPo/ + ; 70A09-GAL4/UAS > STOP > Chrimson.mVenus (70A09 activation). (**b**) Difference in flies mean speed between (**b**) activation-baseline periods for all genotypes and (**c**) lightsOFF-baseline periods for 70A09 activation. (**d**) Copulation of activated and control females with the distribution according to latency to copulation. Number of receptive females out of the total number of females are shown in parenthesis. Statistical analysis was performed with Kruskal–Wallis test (**b**), followed by post hoc pairwise Dunn’s test with Bonferroni correction, Wilcoxon rank-sum test: ns = not significant, **p < 0.01. n values are shown in parentheses.

Silencing 70A09 neurons leads to decreased receptivity, which is accompanied by increased escape (higher walking speed, less pausing and more jumping) during courtship. We sought to explore the effect of activating these neurons during courtship. To this end, we expressed the red shifted channelrhodopsin, csChrimson^42^ in 70A09 neurons. We recorded single pairs of courting flies for 9 min. The red light was off during the first 3 min, it was turned on from minute 3 to 6 and was again off for the last 3 min in order to allow within-video comparisons. In this experiment, we quantified speed which includes pausing and jumping (as opposed to walking speed which does not). We observed that upon light activation the test flies drastically reduced their speed while the speed of control flies was unchanged (Fig. 6a and b). In fact, activated females paused during light on, only performing lateral displacement prompted by the courting male (Movie 1). Once the light was off, activated females recovered their speed to values similar to those prior to activation as shown by comparing the delta of the speed during lights off and baseline to a database with a random group of values with a similar range varying around zero (Fig. 6c). Given that silencing 70A09 neurons reduces receptivity, we wondered what would be the effect of activation of these neurons on receptivity. Analysis of the latency to copulate shows that activated flies did not mate during light on and resume mating once the light turns off whereas control females mate throughout the whole video, indicating that activation of 70A09 neurons leads to a reduction of receptivity (Fig. 6d). Courtship remains high throughout the experiment (Figure S6). It is unclear whether it is activation and silencing of the same or a different set of neurons within the 70A09 expression that leads to loss of receptivity.

With these experiments we found that, in terms of speed, activation of 70A09 neurons leads to the opposite phenotype of silencing them during courtship ON. We speculate that in wild type receptive females these neurons are gradually activated during courtship ultimately leading to female pausing.

## Discussion

Courtship allows animals to display and evaluate their qualities before they choose a mate. In most species, males initiate courtship and females decide whether or not to mate^43^. Reproductive decisions have a powerful impact in the survival of the species and thus the communication between courtship partners is vital. To understand courtship behaviours, we must focus on how the partners communicate which sometimes involves subtle cues.

Here we reveal a novel layer of regulation of female speed in the context of courtship. Specifically, we found that escape suppression is a fundamental and hitherto unknown step of the female’s response to courtship. When modulation of the 70A09 is absent, females escape the courting male continuously and vigorously. One could assume that these females are not able to perceive courtship from the male, perceiving instead an approaching animal, which would lead them to escape. But in fact, these females are recognizing the courtship song and this stimulus is inducing escape. Our work suggests that part of the female brain is interpreting song as aversive while another part processes song as a signal to slow down. We propose that activity in 70A09 brain neurons tips the scale to slowing down. Escape is occasionally observed in wild type receptive virgins, usually early in courtship. We speculate that courtship is initially aversive to the female which with continued courtship is adjusted to an opportunity to mate leading to reducing the speed and eventually accepting the male.

While our work highlights the impact of the acoustic stimuli produced by the male during courtship, it is well established that chemical stimuli such as cis-vaccenyl acetate (cVA) and cuticular hydrocarbons are a major component of the communication between flies and play a role in the female’s decision to mate^44–46^. This work opens the way to investigate how chemical stimuli contribute, in combination with courtship song, to the modulation of female speed during courtship.

Wild type unreceptive flies, i.e., immature virgins and mated females, respond to courtship differently from receptive virgins. Immature virgins do not slow down and pause less than mature virgins^6,17,21^. Mated females do not display high walking speed, as immature females do, but show a positive correlation between the song amount and their speed^6,18^. In sum, some features of the natural unreceptive states are common to 70A09 silencing phenotype indicating that 70A09 neurons may be differently active in receptive and unreceptive females.

Beside a role in escape modulation, we have also uncovered a role of 70A09 neurons in receptivity that is separable from the ability to escape. Though it is clear from wild type behaviour the close link between speed modulation during courtship and receptivity, which 70A09 neuron(s) are involved in the receptivity and the escape phenotype remains to be elucidated.

A recent study characterised neurons in the central brain, pC2l, that are tuned to courtship song and modulate the locomotor response in a sex-specific manner^23^. Though the exact identity of 70A09 neurons which are involved in the observed escape phenotypes is unknown, it is clear that they do not overlap with *dsx*-positive pC2l since we have shown that the *dsx* subset of 70A09 neurons do not show an escape phenotype. Moving forward it would be interesting to investigate how pC2l and 70A09 neurons interact to produce a locomotor response to song.

In conclusion, our work shed a light on the interactions between mating partners, by revealing a new role of the male courtship song and identifying a set of brain neurons responsible for the song-induced female slowing down. The male song is a courtship cue with a dual role and opposite effects on the female: it first induces escape, providing the female with enough time to assess the male, until the decision to mate is made, and then it prompts a decrease in locomotion, which in turns will allow the male to get closer to the female and eventually copulate. The activity in 70A09 neurons is necessary for suppressing the song-induced escape by prompting a decrease in locomotion and allowing to advance the courtship plot. Our findings highlight the complexity of male–female interactions during courtship, revealing a dual response of the female to courtship song.

## Methods

### Fly stocks

Fly strains and sources are as follows: Canton-S (CS), w^1118^ ^47^, GMR70A09-GAL4 and all lines in receptivity screen^30^, 65C12-DBD^48^, UAS-*Kir2.1*^31^, Tub-GAL80TS^32^; *otd-nls*:FLPo^36^, UAS > STOP > *Kir2.1*^34^, UAS > STOP > *CD8-GFP*^49^, *8xLexAop2-FLP*_*L*_^50^, Gad-LexA^51^, elav^GAL4DBD^^52^, ey-FLP^53^, *elav-*GAL80^34^, *Dilp3*-GAL4^54^, TH-DBD^55^ provided by Gerald Rubin, *UAS* > *STOP* > *csChrimson.mVenus*^42^ FLP-out version provided by Vivek Jayaraman, fru^LexA^^56^, fru^GAL4^ ^41^ and dsx^DBD^^57^.

### Construction of transgenic lines

The *70A09*-LexA and *70A09*-AD DNA constructs were generated by Gateway cloning technology (Invitrogen). The entry clone (pCR8/GW/TOPO; Invitrogen) carrying *70A09* enhancer fragment^58^, generously provided by Gerald Rubin, was cloned into pBPLexA::p65Uw (Addgene plasmid #26230) and pBPp65ADZpUw (Addgene plasmid #26234). DNA constructs were verified by restriction enzymatic digestion with *XbaI* (New England Biolabs #R0145) for 2 h at 37 °C and purified using QIAGEN Plasmid Midi Kit (Cat Nº. 12,145), prior to injection into flies. Plasmid was injected into y^1^ w^67c23^; P{CaryP}attP40 flies^59^ by adapting a protocol from Kiehart et al*.*^60^.

### Immunostaining and Microscopy

Adult brains and VNCs were dissected in cold phosphate-buffered saline (PBS), fixed in 4% paraformaldehyde (PFA) in PBL (PBS and 0.12 M Lysine) for 30 min at room-temperature (RT), washed three times for 5 min in PBT (PBS and 0.5% Triton X-100) and blocked for 15 min at RT in 10% Normal Goat Serum (NGS, Sigma-Aldrich) in PBT. Tissues were incubated with the primary antibodies in blocking solution for 72 h at 4 °C. The following primary antibodies were used: rabbit anti-GFP (1:2000, Molecular Probes, cat# A11122), and mouse anti-nc82 (1:10, Developmental Studies Hybridoma Bank). Samples were washed three times for 5 min in PBT and incubated in Alexa Fluor secondary antibodies (1:500, Invitrogen) for 72 h at 4 °C. The following secondary antibodies were used: anti-rabbit IgG conjugated to Alexa 488 and anti-mouse IgG conjugated to Alexa 594. Samples were washed three times for 5 min in PBT and mounted in VECTASHIELD (Vector Laboratories, Cat# H1000). Images were acquired on a ZEISS LSM 710 confocal microscope using 20 × objective or 25 × Immersion objective (ZEISS). After acquisition, color levels were adjusted using Fiji^61^.

### Behavioural experiments

Fly husbandry: Flies were raised in standard cornmeal-agar medium at 25ºC and 70% relative humidity in a 12 h:12 h dark:light cycle, unless otherwise indicated. For all experiments both female and male flies were collected under CO_2_ anesthesia, soon after eclosion, and raised in regular food vials. Flies were raised in isolation for fertility and receptivity experiments. Females were raised in groups of up to 25 per vial for looming experiments. For acute neuronal silencing experiments, female flies and males were raised at 18 °C from 6 to 14 days. Manipulated flies were incubated at 30 °C for 24 h, whereas control flies were maintained at 18 °C. Both controls and manipulated flies, as well as males, were shifted to 25 °C 24 h before the behavioural assay to prevent the effect of temperature treatment on the behaviour. For chronic neuronal silencing, female flies and males were raised at 25 °C from 4 to 8 days.

Unless specified, the flies used in behavioural experiments were 4–8 days old virgin females and males, and were tested in the same conditions as rearing (25 °C and 70% humidity).

1. *Fertility screen*
  To allow mating, a male and a test female were paired in a food vial for 30 min after which the male was removed. One week later the vial was checked for progeny. For each line 20–25 females were tested. The lines for which at least 25% of the females did not produce progeny were selected for further testing. In this initial large-scale screen, controls were not used.
2. *Female receptivity*
  To test female receptivity, a single female was gently aspirated and transferred into circular acrylic chambers (small arenas: 16 mm in diameter × 4.5 mm height) and paired with a male. Individual pairs were recorded for 30 min using Sony HDR-CX570E, HDR-SR10E, HDR-XR520VE or HDR-PJ620 video cameras (1440 × 1080 pixels; 25 frames per second). A white LED was used as backlight source (Edmund Optics, cat# 83-875).
3. *Receptivity with female tracking*
  To allow the detailed analysis of the behaviour, a single female was gently aspirated and transferred to a custom-made circular arena with a conical-shaped bottom that avoid flies walking on the walls^62^ (detailed arenas: 40 mm in diameter), allowing to track them as described in Aranha et al*.*^16^. Each female was allowed to habituate to the new environment for about 10 min and then paired with a male. Movies were acquired in dim light using an infrared 940 nm LED strip (SOLAROX) mounted on an electric board developed by the Scientific Hardware Platform. Flies were recorded in grayscale (1024 × 1024 pixels, 60 frames per second), with a camera mounted above the arena (Point Grey FL3-U3-32S2M-CS with a 5 mm fixed focal length lens (Edmund Optics)) with a HOYA 49 mm R72 infrared filter, for 20 min or until copulation occurred. Female flies paired with *fruitless* mutant males were recorded for 10 min. Bonsai^63^ was used for movie acquisition. To generate wingless males, individual CS male flies were anesthetized with CO_2_ approximately 15–20 h before the experiment. Wings were bilaterally cut at their base with microscissors or microforceps (WPI) under a scope. Flies were allowed to recover at 25ºC until the experiment.
4. *Receptivity in a restricted space*
  To test receptivity in a restricted space, the small arenas were modified by inserting an acrylic adaptor, thus reducing the walking surface (restricted arenas: 6 mm length × 5 mm width × 4.5 height). Single females were gently aspirated and transferred into the restricted arenas. Female flies were allowed to habituate to the new environment for about 10 min before being paired with the male. Movies were acquired in dim light using an infrared 940 nm LED strip (SOLAROX) mounted on an electric board developed by the Scientific Hardware Platform. Flies were recorded for 20 min in grayscale (1024 × 1024 pixels, 60 frames per second), with a camera mounted above the arena (Point Grey FL3-U3-32S2M-CS with a 16 mm fixed focal length lens (Edmund Optics)) with a HOYA 49 mm R72 infrared filter. This setup allowed us to record two pairs of flies at the same time. Bonsai^63^ was used for movie acquisition.
5. *Looming experiment*
  Behavioural apparatus and paradigm: Visual stimulation was delivered on a monitor (Asus ROG Strix XG258Q, 24.5") tilted at 45 degrees over the stage where the arenas were placed. This stage was backlit by an infrared (940 nm) LED array developed by the Scientific Hardware Platform. A 3 mm white opalino was placed between the LED array and the arenas to ensure homogeneous illumination. We recorded behaviour at 60 Hz using a USB3 camera (FLIR Blackfly S, Mono, 1.3MP) with a 730 nm long pass filter (LEE Filters, Polyester 87 Infrared). Behavioural arenas were 30 mm in diameter and 4 mm in height, and were built from opaque white and transparent acrylic sheets. Single flies were transferred to each behavioural chamber using a mouth aspirator. After being transferred, flies were allowed to habituate to the new environment for a period of 2 min. The duration of this baseline period was set based on the median duration that a male takes to start courting the female (latency to court). This baseline period was followed by a stimulation period that lasted 5 min, and during which 7 looming stimuli were presented with an ISI that ranged between 10 and 20 s. Videos were acquired using Bonsai^63^ at 60 Hz and width 1104 × height 1040 resolution.
  Looming stimulus: Looming stimuli were presented on the above-mentioned monitor running at 240 Hz refresh rate; stimuli were generated by a custom Bonsai workflow^63^. The looming effect was generated by a black circle that increased in size over a white background. The visual angle of the expanding circle can be determined by the equation: θ(t) = 2tan − 1 (l / vt), where l is half of the length of the object and v the speed of the object towards the fly. Virtual object length was 1 cm and speed 25 cm s − 1 (l / v value of 40 ms). Each looming presentation lasted for 500 ms. Object expanded during 450 ms until it reached a maximum size of 78° where it remained for 50 ms before disappearing.
6. *Activation experiment*
  For the activation experiment, the female flies were individually collected and allowed to age in cornmeal-agar food containing 0.2 mM all trans-Retinal (Sigma-Aldrich, R2500) and reared in dim light until the experiment.
  The same setup described in the Behavioural Experiment Sect. 3 was used. For the light stimulation a high-powered 610 nm LEDs arrays interspersed between the infrared LEDs on the blacklight board was used. The arena was irradiated with a power in the 4–4.7 mV/cm^2^. A female and a male were gently aspirated and transferred in the arenas. They were allowed to habituate and only when the male started courting the video recording was started. Videos were recorded for 9 min or until copulation. The activation protocol included a baseline that lasts 3 min, followed by light stimulation during 3 min and a post-activation period of 3 min.

### Data processing

In order to quantify female receptivity, a custom-made software was developed to track the flies and compute the time to copulation, when it occurred. To quantify flies’ behaviours, FlyTracker^64^ was used to track the two flies and output information concerning their position, velocity, distance to the other fly, among others. A Courtship Classifier developed in the lab using the machine learning-based system JAABA^65^ was run to automatically identify courtship bouts. Subsequently, in-house developed software PythonVideoAnnotator (https://biodata.pt/python_video_annotator) was used to visualize courtship events generated by JAABA and manually correct them if necessary. Annotations were done from the beginning of courtship and during 10 min or until copulation. PythonVideoAnnotator was also used to manually annotate the copulation time, considering the whole duration of the video.

For the looming experiment, two main features were extracted from the videos using a custom-built Bonsai workflow: centroid position and pixel change in a 72 × 72px ROI around the fly.

### Quantification and statistical analysis

Data analysis was performed using custom Python 3 scripts for all the experiments, except for the copulation rate for small arenas receptivity experiments, for which GraphPad Prism version 7.0 (GraphPad Software) was used. All data, except those from flies excluded due to tracking errors, were analysed.

1. *Female receptivity and male behaviour parameters*
  The latency to copulation was calculated from the beginning of male courtship. With exception of latency to copulation, all quantifications were performed for the first 10 min of courtship or until copulation whichever happened first. Male courtship index was calculated as the ratio between courtship frames and the total number of frames from the beginning of courtship to the end of the video.
2. *Female locomotor parameters during male courtship*
  For the characterisation of female locomotor activity, mean speed, pausing and jumping were quantified. Since courtship is a prerequisite, we selected only videos with courtship index equal or above 20%. The three behaviours were separately quantified in three different moments: (i) before courtship starts (# frames before courtship initiation, (ii) courtship ON (# frames of courtship since courtship initiation) and (iii) courtship OFF (# frames of not courtship since courtship initiation). For the experiment with *fruitless* mutant males, since courtship was absent, the three behaviours were quantified when the distance between the two animals was below 5.5 mm, which is a proxy for courtship, and compared to the same behaviours when the distance was above 5.5 mm. The distance information was extracted from the FlyTracker output (see Data processing section).
  Walking frames were defined as the frames in which female speed was within the range of 4–50 mm/s and the mean walking speed for each fly was calculated by the sum of speed values divided by the number of walking frames. Pausing frames were defined as the frames in which the fly speed was below 4 mm/s, as reported previously^17^. The pausing percentage was obtained normalizing the number of pausing frames over the total number of frames for each courtship moment. Jumps were defined as instantaneous female speed above 70 mm/s. We set this value based on the discontinuity in the speed distribution and on the presence of peaks in the raw, un-binned speed data. Since a high number of peaks were observed for speed values above 50 mm/s (upper limit for walking speed), manual observation of random peaks was performed. Below 70 mm/s most of the peaks corresponded to fly transitions from the lid to the bottom of the arena and/or decamping. Therefore, we set the threshold for jumps at 70 mm/s. For the activation experiment, no speed filter was applied. To observe females’ speed during the whole video recording, rolling average and standard error of the mean (SEM) applied to 15 s were calculated.
3. *Female locomotor parameters during looming stimulus*
  Using the centroid position, a fly was considered to be walking if its speed was higher than 4 mm/s and lower than 75 mm/s. We identified jumping events by detecting peaks in the raw data. A fly was classified as having jumped if its instantaneous speed exceeded a 75 mm/s, a threshold identified by a discontinuity in the speed distribution. The speed plots represent all the moments in which the speed was below the jump threshold for those looming events in which the flies were walking in the 0.5 s bin preceding looming onset, and in the 0.5 s bin from 2.0 to 2.5 s after loom offset.
  For statistical analysis of all experiments, Fisher’s exact test was performed to compare the copulation rate between two different groups. Prior to statistical testing, Levene’s test was used to assess variance homogeneity and Shapiro–Wilk and D'Agostino-Pearson tests were used to assess normality across all individual experiments. Independent groups were subjected to unpaired t-test (n = 2) or one-way ANOVA followed by post hoc pairwise Tukey’s test (n ≥ 3) if parametric assumptions were satisfied. If not, Mann–Whitney U test (n = 2) or Kruskal–Wallis test followed by post hoc Dunn’s test (n ≥ 3) was used. For dependent groups, paired t-test (n = 2) or repeated measures ANOVA followed by post hoc multiple pairwise paired t-test (n ≥ 3) were applied if parametric assumptions were satisfied. If not, Wilcoxon signed-rank test (n = 2) or Friedman’s test followed by post hoc Dunn’s test (n ≥ 3) was used. Bonferroni correction to *p* values was applied when multiple comparisons were performed. Wilcoxon rank-sum test was used to compare one data group with a dataset of random values with median around zero and variance equivalent to the experimental group. The sample size for each condition is indicated in each plot. All the statistical details related to main Figures and Supplementary Figures are included in Tables S2 and S3, respectively. The difference in sample size for the same condition in different analysis is due to the different thresholds applied.

## Supplementary Information

Supplementary Video 1.

Supplementary Information 1.
