# Supplementary material for: Male courtship song drives escape responses that are suppressed for successful mating
Source: Sci Rep. 2021 Apr 29;11:9227. doi: 10.1038/s41598-021-88691-w (PMC8084941; doi:10.1038/s41598-021-88691-w)
Supplement: Supplementary file 2 — Supplementary Information 1. [file 41598_2021_88691_MOESM2_ESM.pdf]

Male courtship song drives escape responses that are suppressed for successful mating

Eliane Arez, Cecilia Mezzera, Ricardo M. Neto-Silva, Márcia M. Aranha, Sophie Dias, Marta A. Moita and Maria Luísa Vasconcelos

Table S1. *Drosophila* female receptivity obtained from a genetic screening of a collection of GAL4 lines. Copulation rate of silencing of GAL4 lines under the control of *TubGal80*^{TS}, with corresponding *p*-values calculated from Fisher's exact statistical test.

Stock #	Janelia ID	Associated gene	N	Copulation index (%)		statistics
				18 ºC	30 ºC	
48068	26H08	Fur1	21	87.5	64.6	p=0.0157 (*)
38693	49E12	5-HT2	24	85.4	85.4	p=1.2265
46027	52D08	GABA-B-R2	25	90.3	94.3	p=0.6595
45362	72B02	DopR	28	95.8	41.7	p<0.0001 (****)
47069	80F08	Dat	20	77.8	78.3	p=1.0000
46831	87B08	RunxB	27	88	82	p=0.5766
47720	70A09	5-HT7	21	72.9	12.5	p<0.0001 (****)
39510	70A08	5-HT7	21	84.1	60	p=0.0176 (*)
46676	72D03	D2R	23	95.8	81.3	p=0.5050
47902	22A08	nAcRalpha-96Aa	23	89.6	91.7	p=1.0000
39462	68B11	Octbeta2R	25	89.4	85.4	p=0.7589
46642	70H01	DopR2	23	87.5	95.8	p=0.2678
38843	52G04	5-HT1A	25	91.7	70.8	p=0.0169 (*)
45490	23E09	GRHRII	31	79.2	89.6	p=0.1631
39464	68C01	Octbeta2R	23	81.3	56.3	p=0.0147 (*)
47673	57G02	nAcRbeta-64B	23	95.8	43.8	p<0.0001 (****)
39544	70F08	Rdl	20	88.9	84.4	p=0.7578
39419	27A07	Fur1	25	95.8	89.6	p=0.4353
39511	70B01	5-HT7	23	100	93.8	p=0.2421
45471	22B06	fru	22	97.9	97.9	p=1.0000
46197	33H05	ct	22	85.4	85.4	p=1.0000
38744	50D04	5-HT2	17	85.1	89.6	p=0.5523
49631	94D02	en	24	68.8	25	p<0.0001 (****)
48043	22H11	fru	24	91.7	95.8	p=0.6773
48977	22C11	fru	24	91.7	2.1	p<0.0001 (****)
49193	26F06	Fur1	20	82.5	82.9	p=1.0000
48979	22D01	nAcRalpha-96Aa	23	91.7	81.3	p=0.2321
49017	23B08	nAcRalpha-30D	25	93.8	97.9	p=0.6170
49019	23B11	fru	22	92.3	92.3	p=1.0000
48992	22F07	nAcRalpha-96Aa	24	93.5	97.8	p=0.6166
49012	23A11	nAcRalpha-96Aa	25	88.4	55.8	p=0.0015 (**)
49021	23C03	fru	25	93.8	83.3	p=0.1986
49143	25H03	lz	29	93.8	50	p<0.0001 (****)
49006	23A03	AlstR	24	91.7	45.8	p<0.0001 (****)
49015	23B02	fru	22	100	93.6	p=0.2419
49175	26D04	Fur1	21	92.3	89.7	p=1.0000
49008	23A05	nAcRalpha-96Aa	25	93.8	85.4	p=0.3167
49602	64D03	Takr99D	24	93.2	97.7	p=0.6162
49926	36A05	ct	24	82.5	93.8	p=0.1753
49559	44H01	lilli	23	89.6	89.6	p=1.0000
49441	27H08	mam	23	91.7	81.3	p=0.2321
49457	28E01	Lmpt	23	91.7	85.1	p=0.3553
49299	22B04	Adar	23	88.9	62.9	p= 0.0130 (*)
39514	70B07	5-HT7	22	83.3	83.3	p=1.0000
49301	22C05	fru	23	92.1	76.3	p=0.1132
49342	29E06	Pkc53E	21	89.1	0	p<0.0001 (****)
49608	64H06	amn	21	87.8	82.9	p=0.7560
46643	70H08	DopR2	24	83.3	76.7	p=0.1505
49494	29F10	fdl	24	79.2	4.2	p<0.0001 (****)
49338	29B09	Pkc53E	14	83.7	82.9	p=1.0000
49944	37A02	nAcRalpha-96Aa	20	92.1	84.2	p=0.4799
49208	27A05	Fur1	25	88.6	86.1	p=1.0000
49459	28E05	Dscam	24	74.4	84.2	p=0.4009
49424	88E07	ems	27	93.8	2.1	p<0.0001 (****)
49428	94D06	en	24	97.2	97.1	p=1.0000
49907	35D04	ct	25	88.6	91.1	p=0.7391
48027	9F02	retn	23	91.5	95.8	p=0.4353
49320	25E10	Adf1	23	94.4	91.2	p=0.6690
48138	40A05	dsx	25	91.7	27.8	p<0.0001 (****)
49494	29F10	fdl	24	79.2	4.2	p<0.0001 (****)
49350	30G04	Nrg	14	81.3	27.1	p<0.0001 (****)
49523	30B11	ple	25	89.6	81.3	p=0.3864
46963	76B10	GABA-B-R3	25	85.4	85.4	p=1.0000
49384	39F10	SoxN	23	83.3	72.9	p=0.3235
46385	57F02	nAcRalpha-30D	24	81.3	50	p=0.0021 (**)

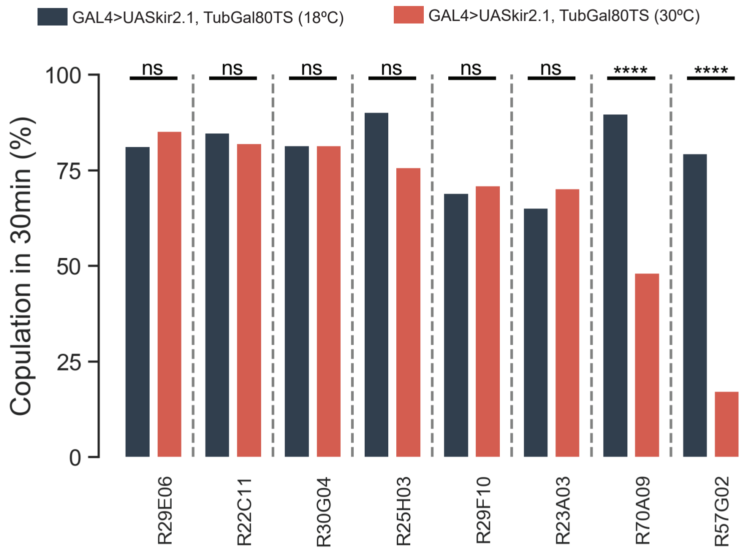

Figure S1 – **Screening of GAL4 lines for receptivity upon silencing of brain neurons**.
Receptivity of virgin females carrying the indicated GAL4 lines and *UAS-Kir2.1*, *TubGal80*^{TS}. Statistical analysis was performed with Fisher's exact test: ns = not significant, **** p < 0.0001. n=40-48.

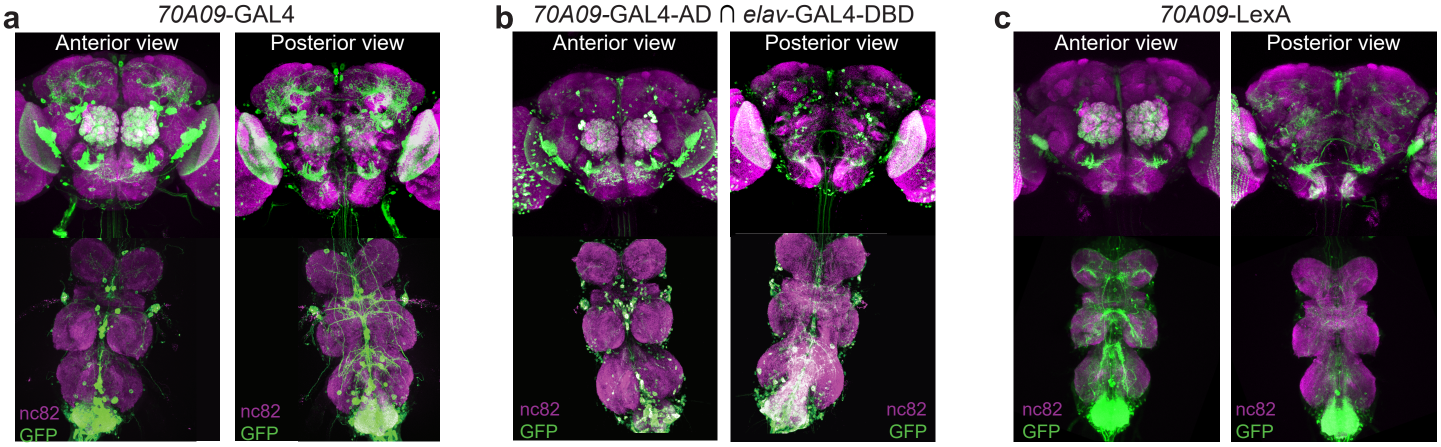

Figure S2 – Anatomical characterisation of three driver lines under the control of *70A09* enhancer.
Confocal images of brains and VNCs from female flies carrying **(a)** *70A09*-GAL4 and *UAS-CD8::GFP*, **(b)** *70A09*-AD ∩ *elav*DBD and *UAS-CD8::GFP*, **(c)** *70A09*-LexA and *LexAop-CD2-GFP*. GAL4, split-GAL4 and LexA-driven expression is shown in green while the synaptic marker nc82 is shown in magenta.

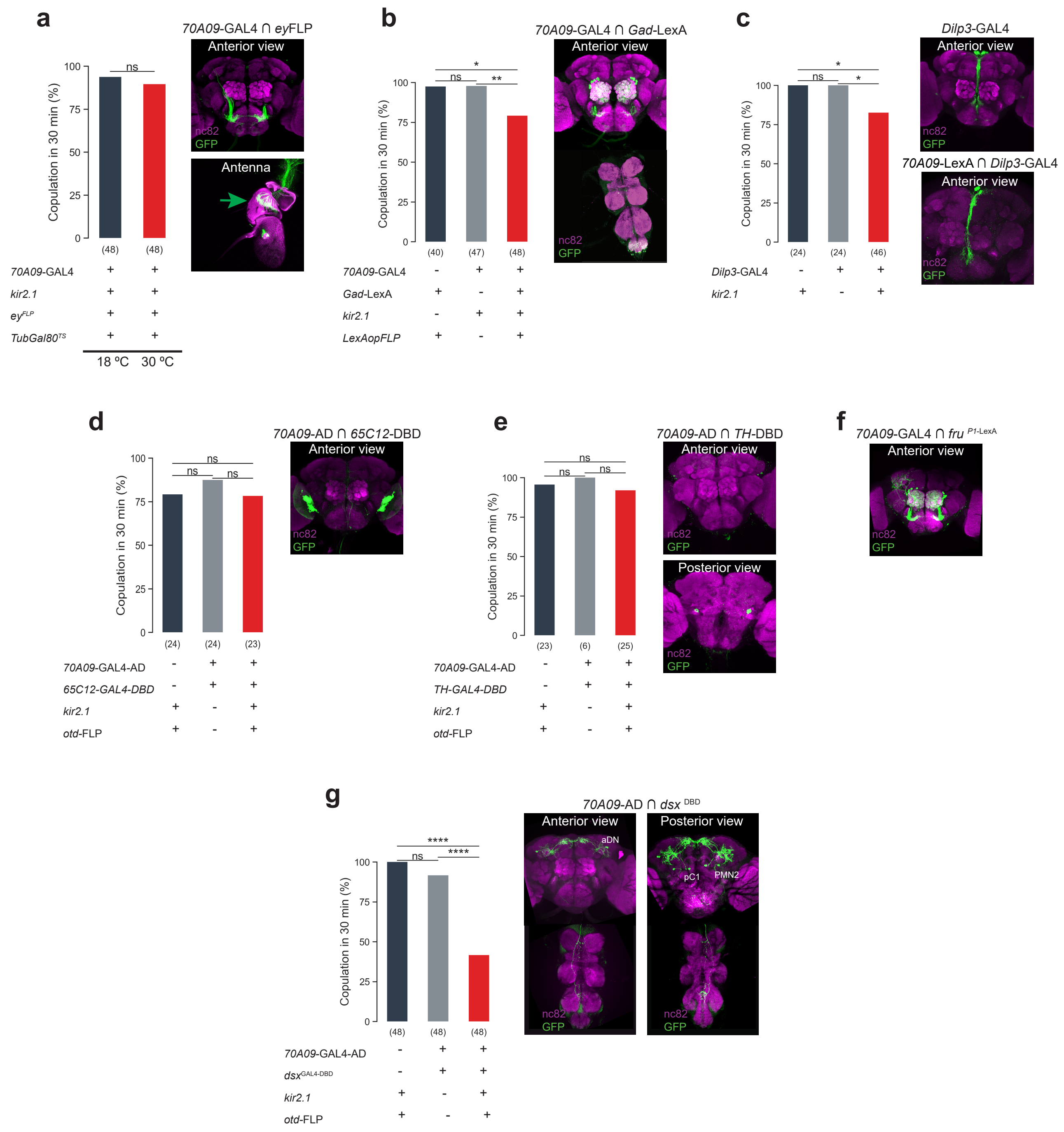

Figure S3 – Female receptivity phenotype upon silencing different subsets of *70A09* neurons.

(a – e) Copulation rate of silenced and control females (left) when silencing different sets of *70A09*-positive neurons shown in confocal images (right). For all the mating analysis statistical analysis were performed with Fisher's exact test: ns = not significant, *p<0.05, **p<0.01. **(a)** Anterior view of female brain (top) and view of the Johnston's organ in the antenna (arrow, bottom) showing sensory neurons obtained from the intersection of *70A09*-GAL4 with *ey*-FLP. **(b)** Anterior view of female brain and VNC showing the expression pattern of *70A09*-GAL4 intersected with *gad*-LexA. **(c)** Anterior view of female brain showing *Dilp3*-GAL4 expression (top) and *70A09*-LexA intersected with *Dilp3*-GAL4 (bottom). For mating experiment only *Dilp3*-GAL4 was used to drive *kir2.1* expression in *ilp3*-expressing neurons. **(d)** Anterior view of female brain showing the LC17 neurons obtained from the intersection of *70A09*-GAL4-AD with 65C12-GAL4-DBD. **(e)** Anterior and posterior views of female brain showing the expression pattern of *70A09*-GAL4-AD intersected with TH-GAL4-DBD. **(f)** Anterior view of female brain showing *70A09-fruitless* positive neurons obtained from the intersection of *70A09*-GAL4 with fru^{P1}-LexA. **(g)** Anterior and posterior views of female brain showing *70A09-doublesex* positive neurons obtained from the intersection of *70A09*-GAL4-AD with $dsx^{GAL4\text{-}DBD}$. For all these confocal images, neurons were visualised with anti-GFP (green) and the tissue counterstained with the synaptic marker nc82 (magenta). n values are shown in parentheses.

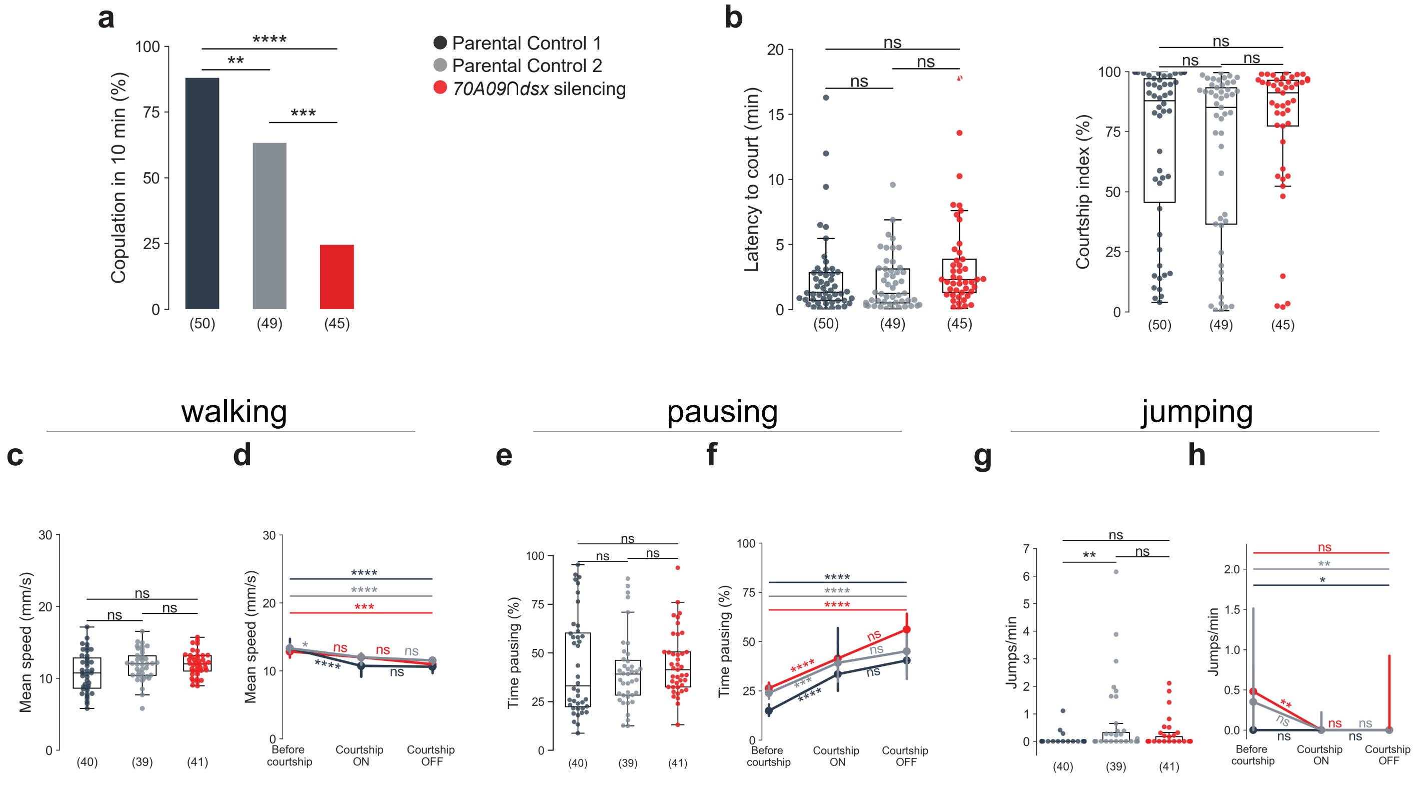

Figure S4 – Copulation, courtship and locomotion behavior of 70A09∩dsx silenced females.

(a) Copulation rate of silenced and control females. Genotypes: w-/UAS>STOP>kir2.1; otd-nls:FLPo/+; + (Parental Control 1), w-; 70A09-GAL4-AD/+; dsxGAL4-DBD/+ (Parental Control 2) and w-/UAS>STOP>kir2.1; 70A09-GAL4-AD/otd-nls:FLPo; dsxGAL4-DBD/+ (70A09∩dsx silencing). **(b)** Male latency to court (left) and courtship index toward silenced and control females (right). **(c)** Female mean walking speed (4 – 50 mm/s), **(e)** female pausing and **(g)** number of jumps per minute, during courtship ON periods. **(d)** Female mean walking speed (4 – 50 mm/s), **(f)** percentage of time females spend pausing and **(h)** number of jumps per minute, in different moments of courtship dynamics. Statistical analysis was performed with Fisher's exact test **(a)**, Kruskal-Wallis **(b, e, g)** and Friedman's test **(d:** 70A09∩dsx silencing**, f and h)** followed by post hoc Dunn's test with Bonferroni correction, one-way ANOVA followed by post hoc Tukey's test **(c)**, repeated measures ANOVA followed by post hoc multiple pairwise paired t-test with Bonferroni correction **(d:** parental controls**)**: ns = not significant, *p<0.05, **p<0.01, ***p<0.001, ****p<0.0001. n values are shown in parentheses.

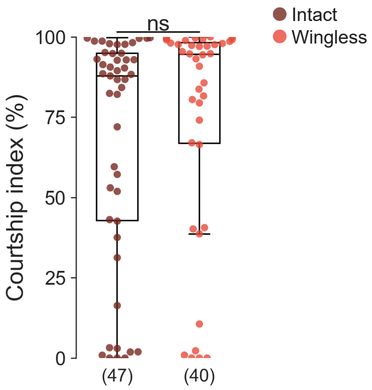

Figure S5 – **Courtship index of wingless and intact males.**
Male courtship index of intact males and males without wings toward *70A09* silenced females. Genotype: w-*/UAS>STOP>kir2.1*; *otd*-nls:FLPo/+; *70A09*-GAL4/+. Statistical analysis was performed with Mann–Whitney U test: ns = not significant. n values shown in parentheses.

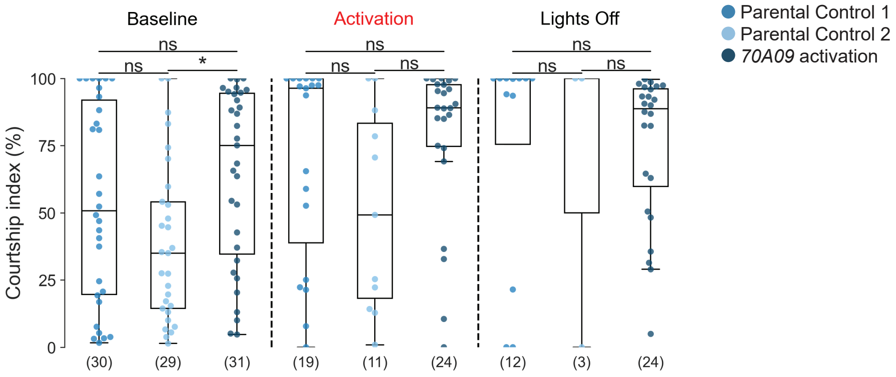

Figure S6 – **Male courtship in each period of the activation experiment.**
Male courtship index toward activated and control females, for each moment of the activation experiment. Genotypes: *w*-; *otd*-nls:FLPo/+; *UAS>STOP>Chrimson.mVenus* (Parental Control 1), *w-; +; 70A09*-GAL4/+ (Parental Control 2) and w-; *otd*-nls:FLPo/+; *70A09*-GAL4/*UAS>STOP>Chrimson.mVenus* (*70A09* activation). Statistical analysis was performed with Kruskal-Wallis test, followed by post hoc Dunn's test with Bonferroni correction: ns = not significant, *p<0.05. n values are shown in parentheses.

Table S2. Statistical details related to Main Figures.

Figure	groups	n	normally distributed	statistical test	p value	dfs	
1b	a) UAS>STOP>Kir2.1; otd-FLP; 70A09GAL4/elavGAL80	49	NA	Fisher's exact test	a vs b = 0,0291; a vs c = 0,3011; a vs d = 0,0000	3	
	b) UAS>STOP>Kir2.1; ; 70A09GAL4	35	NA				
	c) ; otd-FLP; elavGal80	35	NA				
	d) UAS>STOP>Kir2.1; otd-FLP; 70A09GAL4	49	NA				
2b	a) parental control 1	45	no	Kruskal-Wallis test with post hoc Dunn's test	a vs b = 1,0000; a vs c = 1,0000; b vs c = 0,2765. H=2,85	2	a vs b: 88
	b) parental control 2	45	no				a vs c: 92
	c) 70A09 silencing	49	no				b vs c: 92
2c	a) parental control 1	45	NA	Fisher's exact test	a vs b = 1,0000; a vs c = 0,0000; b vs c = 0,0000	2	
	b) parental control 2	45	NA				
	c) 70A09 silencing	49	NA				
2d	a) parental control 1	45	no	Kruskal-Wallis test with post hoc Dunn's test	a vs b = 1,0000; a vs c = 0,9978; b vs c = 1,0000. H=0,95	2	a vs b: 88
	b) parental control 2	45	no				a vs c: 92
	c) 70A09 silencing	49	no				b vs c: 92
2f	a) parental control 1	39	no	Kruskal-Wallis test with post hoc Dunn's test	a vs b = 1,0000; a vs c = 1,14E-09; b vs c = 2,13E-11. H=58,62	2	a vs b: 78
	b) parental control 2	41	no				a vs c: 81
	c) 70A09 silencing	44	no				b vs c: 83
2g	a) parental control 1	35	no	Friedman test with post hoc Dunn's test	before vs ON = 0,0005; before vs OFF = 4,00E-05; ON vs OFF = 1,0000. Q=36,40	2	68
	b) parental control 2	39	yes	rmANOVA with post hoc mpPaired t-test	before vs ON = 3,00E-06; before vs OFF = 3,32E-07; ON vs OFF = 1,0000. F=45,03	2	76
	c) 70A09 silencing	43	no	Friedman test with post hoc Dunn's test	before vs ON = 5,60E-05; before vs OFF = 0,9674; ON vs OFF = 0,0030. Q=21,91	2	84
2h	a) parental control 1	39	no	Kruskal-Wallis test with post hoc Dunn's test	a vs b = 1,0000; a vs c = 0,0008; b vs c = 0,0003. H=19,32	2	a vs b: 78
	b) parental control 2	41	no				a vs c: 81
	c) 70A09 silencing	44	no				b vs c: 83
2i	a) parental control 1	38	no	Friedman test with post hoc Dunn's test	before vs ON = 5,00E-06; before vs OFF = 3,50E-05; ON vs OFF =1,0000. Q=33,21	2	74
	b) parental control 2	41	no		before vs ON = 1,00E-06; before vs OFF = 2,16E-08; ON vs OFF = 1,0000. Q=48,20	2	80
	c) 70A09 silencing	43	no		before vs ON = 1,0000; before vs OFF = 3,00E-05; ON vs OFF = 7.70E--05. Q=34,09	2	84
2j	a) parental control 1	39	no	Kruskal-Wallis test with post hoc Dunn's test	a vs b = 0,8379; a vs c = 7,66E-08; b vs c = 1,78E-05. H=35,57	2	a vs b: 78
	b) parental control 2	41	no				a vs c: 81
	c) 70A09 silencing	44	no				b vs c: 83
2k	a) parental control 1	39	no	Friedman test with post hoc Dunn's test	before vs ON = 0,6325; before vs OFF = 0,1860; ON vs OFF = 1,0000. Q=3,50	2	76
	b) parental control 2	41	no		before vs ON = 1,0000; before vs OFF = 1,0000; ON vs OFF = 1,0000. Q=0,40	2	80
	c) 70A09 silencing	44	no		before vs ON = 3,00E-06; before vs OFF = 0,0123; ON vs OFF = 0,1328. Q=24,00	2	86
3c	a) parental control 1	463	no	Kruskal-Wallis test with post hoc Dunn's test	a vs b = 0,1893; a vs c = 2,78E-07; b vs c = 0,0021. H=31,09	2	a vs b: 931
	b) parental control 2	470	no				a vs c: 1071
	c) 70A09 silencing	610	no				b vs c: 1078
3d	a) baseline, parental control 1	140	no	Kruskal-Wallis test with post hoc Dunn's test	a vs b = 1,0000; a vs c = 4,99E-17; b vs c = 3,36E-16. H=94,78	2	a vs b: 277
	b) baseline, parental control 2	139	no				a vs c: 278
	c) baseline, 70A09 silencing	140	no				b vs c: 277
3d	a) looming, parental control 1	140	no	Kruskal-Wallis test with post hoc Dunn's test	a vs b = 0,0020; a vs c = 0,0036; b vs c = 9,93E-11. H=44,20	2	a vs b: 277
	b) looming, parental control 2	139	no				a vs c: 278
	c) looming, 70A09 silencing	140	no				b vs c: 277
3e	a) parental control 1	14	no	Kruskal-Wallis test with post hoc Dunn's test	a vs b = 0,0019; a vs c = 1,0000; b vs c = 0,0253. H=12,83	2	a vs b: 29
	b) parental control 2	17	no				a vs c: 44
	c) 70A09 silencing	32	no				b vs c: 47
4b	a) parental control 1	14	no	Kruskal-Wallis test with post hoc Dunn's test	a vs b = 1,0000; a vs c = 1,0000; b vs c = 0,9758. H=0,98	2	a vs b: 29
	b) parental control 2	17	no				a vs c: 44
	c) 70A09 silencing	32	no				b vs c: 47
4c	a) parental control 1	21	NA	Fisher's exact test	a vs b = 0,5192; a vs c = 0,0000; b vs c = 0,0000	2	
	b) parental control 2	24	NA				
	c) 70A09 silencing	47	NA				
4d	a) parental control 1	21	no	Kruskal-Wallis test with post hoc Dunn's test	a vs b = 1,0000; a vs c = 0,2886; b vs c = 0,1010. H=5,58	2	a vs b: 43
	b) parental control 2	24	no				a vs c: 66
	c) 70A09 silencing	47	no				b vs c: 69
5a	a) parental control 1	26	yes	one-way Anova with post hoc Tukey's HSD test	a vs b = 0,1304; a vs c = 0,9000; b vs c = 0,1722. F=2.39	2	a vs b: 51
	b) parental control 2	27	yes				a vs c: 50
	c) 70A09 silencing	26	yes				b vs c: 51
5b	a) parental control 1	26	yes	paired t-test	≤ 0.5mm vs 1 > 0.5mm = 0,0003; t= -4,16	50	
	b) parental control 2	27	yes		≤ 0.5mm vs 1 > 0.5mm = 0,0006; t= -3,93	52	
	c) 70A09 silencing	26	yes		≤ 0.5mm vs 1 > 0.5mm = 0,0004, t= -4,07	50	
5c	a) parental control 1	26	no	Kruskal-Wallis test with post hoc Dunn's test	a vs b = 0,0032; a vs c = 0,0094; b vs c = 1,0000. H=12,96	2	a vs b: 51
	b) parental control 2	27	no				a vs c: 50
	c) 70A09 silencing	26	no				b vs c: 51
5d	a) parental control 1	26	no	Wilcoxon signed rank test	≤ 0.5mm vs 1 > 0.5mm = 1,33E-05; w = 4,0	50	
	b) parental control 2	27	no		≤ 0.5mm vs 1 > 0.5mm = 1,70E-05; w = 10,0	52	
	c) 70A09 silencing	26	no		≤ 0.5mm vs 1 > 0.5mm = 3,29E-05, w = 12,0	50	
5e	a) parental control 1	26	no	Kruskal-Wallis test with post hoc Dunn's test	a vs b = 1,0000; a vs c = 1,0000; b vs c = 0,8879. H=1,34	2	a vs b: 51
	b) parental control 2	27	no				a vs c: 50
	c) 70A09 silencing	26	no				b vs c: 51
5f	a) parental control 1	26	no	Wilcoxon signed rank test	≤ 0.5mm vs 1 > 0.5mm = 0,4990; w = 10,0	50	
	b) parental control 2	27	no		≤ 0.5mm vs 1 > 0.5mm = 0,1614; w = 8,0	52	
	c) 70A09 silencing	26	no		≤ 0.5mm vs 1 > 0.5mm = 0,8590; w = 21,0	50	
5g	a) intact	38	yes	t- test	a vs b = 3,60E-05; t= -4,41	70	
	b) wingless	34	yes				
5h	a) intact	36	no	Friedman test with post hoc Dunn's test	before vs ON = 0,1198; before vs OFF = 0,0202; ON vs OFF = 6,00E-06. Q=27,72	2	70
	b) wingless	28	no		before vs ON = 1,0000; before vs OFF = 0,0720; ON vs OFF = 0,5228. Q=10,50	2	54
5i	a) intact	38	no	Mann-Whitney U test	a vs b = 2,39E-05; U=285,00	70	
	b) wingless	34	no				
5j	a) intact	34	no	Friedman test with post hoc Dunn's test	before vs ON = 0,2958; before vs OFF = 0,0428; ON vs OFF = 0,0001. Q=21,94	2	66
	b) wingless	29	no		before vs ON = 0,5396; before vs OFF = 0,0064; ON vs OFF = 0,2503. Q=10,83	2	56
5k	a) intact	38	no	Mann-Whitney U test	a vs b = 0,0022; U=402,50	70	
	b) wingless	34	no				
5l	a) intact	32	no	Friedman test with post hoc Dunn's test	before vs ON = 0,0271; before vs OFF = 0,6068; ON vs OFF = 0,5444. Q=4,61	2	62
	b) wingless	36	no		before vs ON = 1,0000; before vs OFF = 1,0000; ON vs OFF = 1,0000. Q=1,46	2	70
6b	a) parental control 1	18	no	Kruskal-Wallis test with post hoc Dunn's test	a vs b = 1,0000; a vs c = 0,0024; b vs c = 0,0014. H = 17,84	2	a vs b: 24
	b) parental control 2	8	no				a vs c: 41
	c) 70A09 activation	25	no				b vs c: 31

6c	a) 70A09 activation<br>b) generated dataset*	22<br>22	no<br>no	Wilcoxon rank-sum test	a vs b = 0,3359; w = -0,96	42	
6d	a) parental control 1<br>b) parental control 2<br>c) 70A09 activation	30<br>29<br>31	no<br>no<br>no	Kruskal-Wallis test with post hoc Dunn's test	a vs b = 0,1726; a vs c = 1,0000; b vs c = 0,0571. H = 6,60	2	a vs b: 57<br>a vs c: 59<br>b vs c: 58

NA: not applicable

rmANOVA: repeated measures ANOVA

mpPaired t-test: multiple pairwise paired t-test

* The generated dataset is a dataset of random values with median around zero and variance equivalent to the experimental dataset

Table S3. Statistical details related to Supplementary Figures.

Figure	groups	n	normally distributed	statistical test	p value	dfs	
S1	R29E06	40	NA	Fisher's exact test	18 C vs 30 C = 0,1957	1	
	R22C11	44	NA	Fisher's exact test	18 C vs 30 C = 0,2140	1	
	R30G04	48	NA	Fisher's exact test	18 C vs 30 C = 0,2056	1	
	R25H03	40	NA	Fisher's exact test	18 C vs 30 C = 0,0741	1	
	R29F10	48	NA	Fisher's exact test	18 C vs 30 C = 0,1715	1	
	R23A03	40	NA	Fisher's exact test	18 C vs 30 C = 0,1687	1	
	R70A09	48	NA	Fisher's exact test	18 C vs 30 C = 7,72E-06	1	
	R57G02	48	NA	Fisher's exact test	18 C vs 30 C = 3,77E-08	1	
S3a	UAS>STOP>kir2.1/eyFLP;+;70A09GA L4/TubGal80TS	48	NA	Fisher's exact test	18 C vs 30 C = 0,7145	1	
S3b	a) w-;8xLexAop2FLP;Gad-LexA	40	NA	Fisher's exact test	a vs b = 1,0000; a vs c = 0,0102; b vs c = 0,0076	2	
	GAL4	47	NA				
	Kir2.1	48	NA				
S3c	a) w-; UASKir2.1; +	24	NA	Fisher's exact test	a vs b = 1,0000; a vs c = 0,0442; b vs c = 0,0442	2	
	b) w-; Dilp3 GAL4; +	24	NA				
	c) Dilp3 GAL4>Kir2.1	48	NA				
S3d	a) UAS>STOP>Kir2.1;otd-FLP;	24	NA	Fisher's exact test	a vs b = 0,7008; a vs c = 1,0000; b vs c = 0,4614	2	
	b) ;70A09-AD;65C12-DBD	24	NA				
	c) otd-FLP ∩ 70A09-AD ∩ 65C12-DBD > Kir2.1	23	NA				
S3e	a) UAS>STOP>Kir2.1;otd-FLP;	23	NA	Fisher's exact test	a vs b = 1,0000; a vs c = 1,0000; b vs c = 1,0000	2	
	b) ;70A09-AD;TH-DBD	6	NA				
	c) otd-FLP ∩ 70A09-AD ∩ 65C12-DBD > Kir2.1	25	NA				
S3g	a) UAS>STOP>Kir2.1;otd-FLP;	48	NA	Fisher's exact test	a vs b = 0,1171; a vs c = 0,0000; b vs c = 0,0000	2	
	b) ;70A09-AD;Dsx^{DBD}	48	NA				
	c) otd-FLP ∩ 70A09-AD ∩ Dsx^{DBD} >	48	NA				
S4a	a) parental control 1	50	NA	Fisher's exact test	a vs b = 0,0050; a vs c = 0,0000; b vs c = 0,0002	2	
	b) parental control 2	49	NA				
	c) 70A09 ∩ Dsx silencing	45	NA				
S4b	a) Courtship latency, parental control 1	50	no	Kruskal-Wallis test with post hoc Dunn's test	a vs b = 1,0000; a vs c = 0,1231; b vs c = 0,0761. H = 6,05	2	a vs b: 97
	b) Courtship latency, parental control 2	49	no				a vs c: 93
	c) Courtship latency, 70A09 ∩ Dsx silen	45	no				b vs c: 92
	a) Courtship index, parental control 1	50	no	Kruskal-Wallis test with post hoc Dunn's test	a vs b = 0,6554; a vs c = 1,0000; b vs c = 0,2101. H = 3,43	2	a vs b: 97
	b) Courtship index, parental control 2	49	no				a vs c: 93
	c) Courtship index, 70A09 ∩ Dsx silenci	45	no				b vs c: 92
S4c	a) parental control 1	40	yes	one-way Anova with post hoc Tukey's HSD test	a vs b = 0,1140; a vs c = 0,0533; b vs c = 0,9000. F = 3,24	2	a vs b: 77
	b) parental control 2	39	yes				a vs c: 79
	c) 70A09 ∩ Dsx silencing	41	yes				b vs c: 78
S4d	a) parental control 1	35	yes	rmANOVA with post hoc mpPaired t-test	before vs ON = 3,00E-05; before vs OFF =1,00E-06; ON vs OFF =1,0000. F = 36,60	2	68
	b) parental control 2	39	yes		before vs ON =0,0131; before vs OFF = 5,10E-05; ON vs OFF = 0,3609. F = 21,19	2	76
	c) 70A09 ∩ Dsx silencing	41	no	Friedman test with post hoc Dunn's test	before vs ON = 0,0625; before vs OFF = 0,0002; ON vs OFF = 0,2741. Q = 32,63	2	80
S4e	a) parental control 1	40	no	Kruskal-Wallis test with post hoc Dunn's test	a vs b = 1,0000; a vs c = 0,7043; b vs c = 0,6954. H = 1,91	2	a vs b: 77
	b) parental control 2	39	no				a vs c: 79
	c) 70A09 ∩ Dsx silencing	41	no				b vs c: 78
S4f	a) parental control 1	38	no	Friedman test with post hoc Dunn's test	before vs ON =7,79E-08; before vs OFF =5,48E-10; ON vs OFF =1,0000. Q = 48,21	2	74
	b) parental control 2	39	no		before vs ON =0,0001; before vs OFF = 1,40E-05; ON vs OFF = 1,0000. Q = 25,08	2	76
	c) 70A09 ∩ Dsx silencing	41	no		before vs ON = 7,00E-06; before vs OFF = 1,64E-10; ON vs OFF = 0,2000. Q = 46,24	2	80
S4g	a) parental control 1	40	no	Kruskal-Wallis test with post hoc Dunn's test	a vs b = 0,0033; a vs c = 0,1574; b vs c = 0,5279. H = 10,76	2	a vs b: 77
	b) parental control 2	39	no				a vs c: 79
	c) 70A09 ∩ Dsx silencing	41	no				b vs c: 78
S4h	a) parental control 1	39	no	Friedman test with post hoc Dunn's test	before vs ON = 0,1870; before vs OFF =0,0324; ON vs OFF =1,0000. Q = 7,00	2	76
	b) parental control 2	39	no		before vs ON =0,0512; before vs OFF = 0,0057; ON vs OFF = 1,0000. Q = 11,94	2	76
	c) 70A09 ∩ Dsx silencing	41	no		before vs ON = 0,0027; before vs OFF = 0,4346; ON vs OFF = 0,1882. Q = 11,38	2	80
S5	a) intact	47	no	Mann- Whitney U test	a vs b = 0,0680; U = 764,50	85	
	b) wingless	40	no				
S6	a) baseline, parental control 1	30	no	Kruskal-Wallis test with post hoc Dunn's test	a vs b = 0,2853; a vs c = 0,9287; b vs c = 0,0215. H = 7,35	2	a vs b: 57
	b) baseline, parental control 2	29	no				a vs c: 59
	c) baseline, 70A09 activation	31	no				b vs c: 58
	a) activation, parental control 1	19	no	Kruskal-Wallis test with post hoc Dunn's test	a vs b = 0,3490; a vs c = 1,0000; b vs c = 0,3119. H = 3,08	2	a vs b: 28
	b) activation, parental control 2	11	no				a vs c: 41
	c) activation, 70A09 activation	24	no				b vs c: 33
	a) light OFF, parental control 1	12	no	Kruskal-Wallis test with post hoc Dunn's test	a vs b = 1,0000; a vs c = 0,1566; b vs c = 0,8825. H = 4,24	2	a vs b: 13
	b) light OFF, parental control 2	3	no				a vs c: 34
	c) light OFF, 70A09 activation	24	no				b vs c: 25

NA: not applicable

rmANOVA: repeated measures ANOVA

mpPaired t-test: multiple pairwise paired t-test
